# Dual impact of EZH1/2-targeted therapy in HTLV-1-associated inflammation: Direct and indirect mechanisms

**DOI:** 10.1016/j.isci.2026.117775

**Published:** 2026-10-09

**Authors:** Yaru Zou, Koju Kamoi, Mingming Yang, Yuan Zong, Jing Zhang, Kyoko Ohno-Matsui

**Affiliations:** 1 Department of Ophthalmology & Visual Science, Graduate School of Medical and Dental Sciences, Institute of Science Tokyo, Tokyo 113-8519, Japan

**Keywords:** HTLV-1, HTLV-1-associated uveitis, EZH1/2 inhibition, valemetostat, epigenetic regulation, H3K27me3, retinal pigment epithelial cells, NF-κB signaling

## Abstract

Human T cell leukemia virus type 1 (HTLV-1)-associated inflammation, including uveitis, lacks effective targeted therapies. Here, we investigated whether epigenetic modulation through EZH1/2 inhibition could influence HTLV-1-related ocular inflammation using *in vitro* co-culture and infection models. HTLV-1 exposure increased EZH1 and EZH2 expression and the repressive histone mark H3K27me3 in retinal pigment epithelial cells. Treatment with the dual EZH1/2 inhibitor valemetostat reduced H3K27me3 levels. Valemetostat showed limited effects on cell viability and induced only modest, context-dependent apoptosis. Virological analyses showed model-dependent changes in proviral load, accompanied by reduced viral gene expression. Profiling of cytokines and chemokines revealed context-dependent modulation of inflammatory mediators. Functional assessment using p65 nuclear translocation assays indicated partial attenuation of NF-κB activation under inflammatory stimulation. Collectively, these findings suggest that EZH1/2-mediated epigenetic regulation is primarily involved in HTLV-1-related inflammatory responses, and that inhibiting EZH1/2 may be a potential approach to modulate inflammatory signaling.

## Highlights

• HTLV-1 exposure upregulates EZH1/2 and H3K27me3 in retinal pigment epithelial cells
• Valemetostat reduces H3K27me3 in HTLV-1-exposed ocular models
• Valemetostat directly lowers proviral load and transcription in infected T cells
• Valemetostat indirectly modulates inflammatory signaling in HTLV-1-associated models

## Graphical abstract

**Figure:**
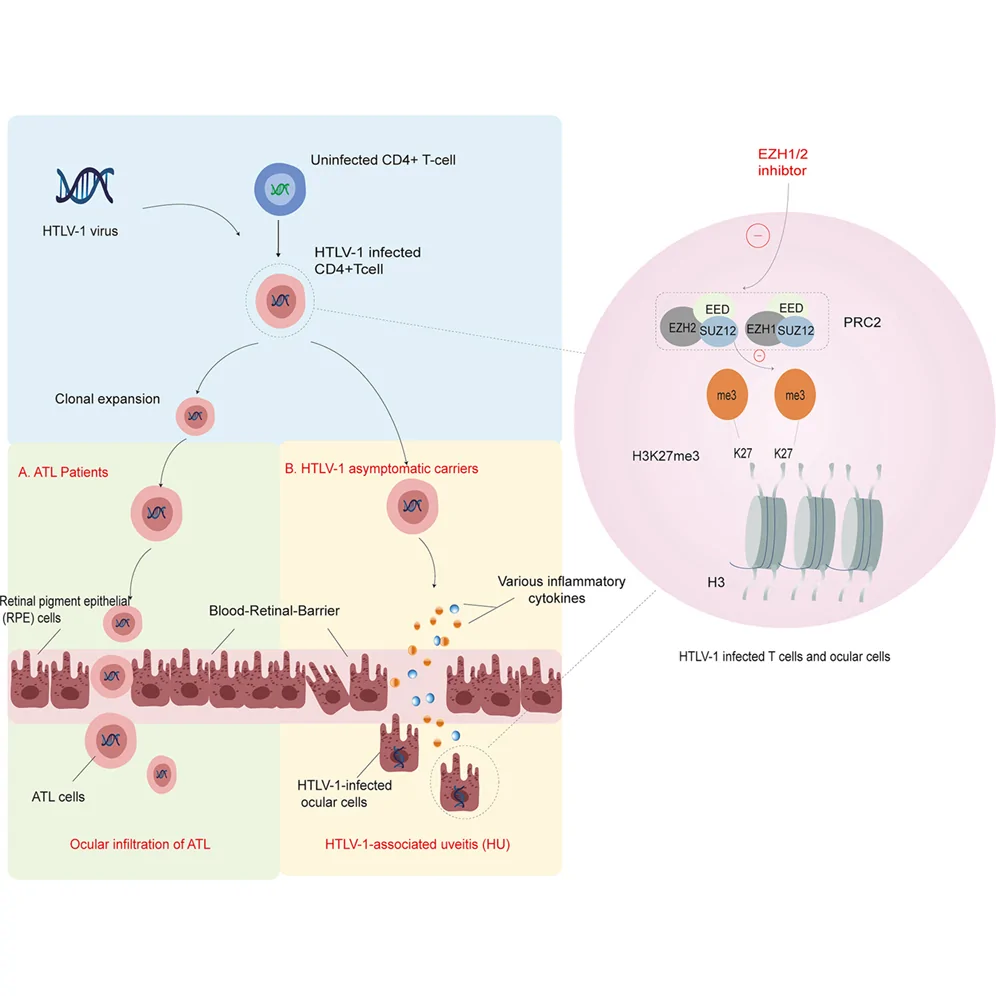

## Teaser

Biological sciences; Molecular biology; Cell biology

## Introduction

Human T cell leukemia virus type 1 (HTLV-1) is the first identified human retrovirus and causes lifelong infection.^1^ An estimated 5–10 million people are infected worldwide, with this figure being only extrapolated from 1/7 of the world’s population.^2^^,^^3^ However, no effective intervention strategies have been actively pursued in decades. Consequently, HTLV-1 poses a serious threat to individual and community health. In recent years, the accelerated pace of human migration has exacerbated this threat, making it a global health concern.^4^ In order to implement more coordinated global public health actions to curb this infectious disease, the World Health Organization (WHO) published relevant technical reports in 2021 to reduce the morbidity and mortality.^5^

HTLV-1 is an RNA virus that uses its RNA-dependent DNA polymerase to generate proviral DNA, which integrates into host T cell genomes.^6^ This lifelong integration contributes to a spectrum of HTLV-1-associated diseases, including adult T cell leukemia/lymphoma (ATL),^7^ HTLV-1-associated uveitis (HU),^8^^,^^9^^,^^10^^,^^11^ and HTLV-1-associated myelopathy (HAM).^12^ Among these, HU is particularly common in endemic regions such as Japan and is characterized by chronic intraocular inflammation that requires long-term ophthalmologic management.^13^

Clinically, intraocular inflammation^14^^,^^15^ can be controlled with topical eye drops and/or intraocular injections of corticosteroids and/or oral prednisone.^16^ However, corticosteroid therapy presents several issues. Firstly, studies have shown that 30–60% of HU patients may experience recurrent uveitis.^17^ Furthermore, various ocular complications may occur after treatment, including cataracts, glaucoma, and macular edema, as well as epiretinal membrane, pigment deposition retinal degeneration, optic nerve atrophy, and bullous keratopathy.^18^ More importantly, corticosteroids cannot completely reduce the number of infected cells or adequately suppress the progression of patient symptoms.^19^ Therefore, there is an urgent need to develop and explore new drugs.

Recent efforts in HTLV-1 research have focused on molecular targeted therapies, including the anti-CCR4 antibody mogamulizumab^20^ and the immunomodulatory agent lenalidomide.^21^ However, treatment-related inflammation and adverse effects remain challenges.^22^ Given the key role of epigenetic dysregulation in HTLV-1-associated diseases, epigenetic therapies have gained increasing attention. In 2022, valemetostat (EZHARMIA), a dual EZH1/2 inhibitor, was approved for the treatment of ATL. EZH2 and its closely related homolog EZH1 are functional enzyme components of the polycomb repressive complex 2 (PRC2) and are often found to be aberrantly expressed in HTLV-1-related conditions; they suppress gene transcription by catalyzing the trimethylation of histone H3 lysine 27 (H3K27).^23^ Previous studies have demonstrated the overexpression of EZH1 and EZH2, as well as increased methylation of H3K27me3 in ATL cells and Tax-expressing cells.^23^ Subsequent studies have also found that dual inhibitors of EZH1/2 can directly inhibit the spontaneous proliferation and the hyper-immune response in HTLV-1-infected cells in patients with HAM.^24^ There are also studies demonstrating that treatment of ATL cell lines with DNA demethylating agents can inhibit cell growth.^25^ These findings suggest that EZH1/2 inhibition may overcome the limitation of corticosteroids, making it a promising potential treatment for patients with HU. However, their potential role in HU remains unexplored.

To address this gap, the present study examined the effects of the dual EZH1/2 inhibitor valemetostat (DS-3201) in HU-related *in vitro* models using both ocular cells and HTLV-1-infected T cell lines.^26^ Adult retinal pigment epithelial (ARPE-19) cells were selected as the ocular model because RPE constitutes the outer blood-retinal barrier and plays a central role in regulating retinal immune homeostasis, which is relevant to the development of ocular inflammation including HU. MT-2 cells (HTLV-1 Tax-positive; transcriptionally active), TL-Om1 cells (HTLV-1-infected ATL-derived; Tax-low/negative and less transcriptionally active), and Jurkat cells (HTLV-1-negative control) were used to represent distinct viral states and biological contexts. Collectively, by investigating the cellular and molecular responses to valemetostat across these models, this study aims to provide insight into potential therapeutic avenues for HTLV-1-associated conditions, particularly HU.

## Results

### EZH1, EZH2, and H3K27me3 are upregulated in HTLV-1-infected ARPE-19 cells

The PRC2 complex, composed mainly of EZH1 and EZH2, catalyzes H3K27 methylation and suppresses the transcription of genes involved in cell-cycle regulation and immune responses. Dysregulation of PRC2 components has been implicated in hematologic malignancies such as ATL and inflammatory disorders including HU.

To investigate whether HTLV-1 infection induces PRC2 activity, we performed western blot and RT-qPCR analyses to quantify the expression of EZH1, EZH2, and H3K27me3 in HTLV-1-infected ARPE-19 cells, HTLV-1-infected MT-2 cells, and uninfected ARPE-19 controls. RT-qPCR showed the upregulation of EZH1 and EZH2 mRNA levels in both HTLV-1-infected ARPE-19 cells and MT-2 cells (Figure 1A). Consistently, western blot analysis revealed markedly higher EZH1 and EZH2 protein expression in these two HTLV-1-positive models compared to ARPE-19 controls (Figure 1B). Statistical analysis confirmed that EZH1 levels in infected ARPE-19 cells were approximately 2.5-fold higher than in the controls (*p* < 0.01), while MT-2 cells exhibited a 6-fold increase (*p* < 0.0001). Similarly, EZH2 expression was elevated in HTLV-1-infected ARPE-19 cells (*p* < 0.01) and MT-2 cells (*p* < 0.0001) (Figure 1C).

**Figure 1:**
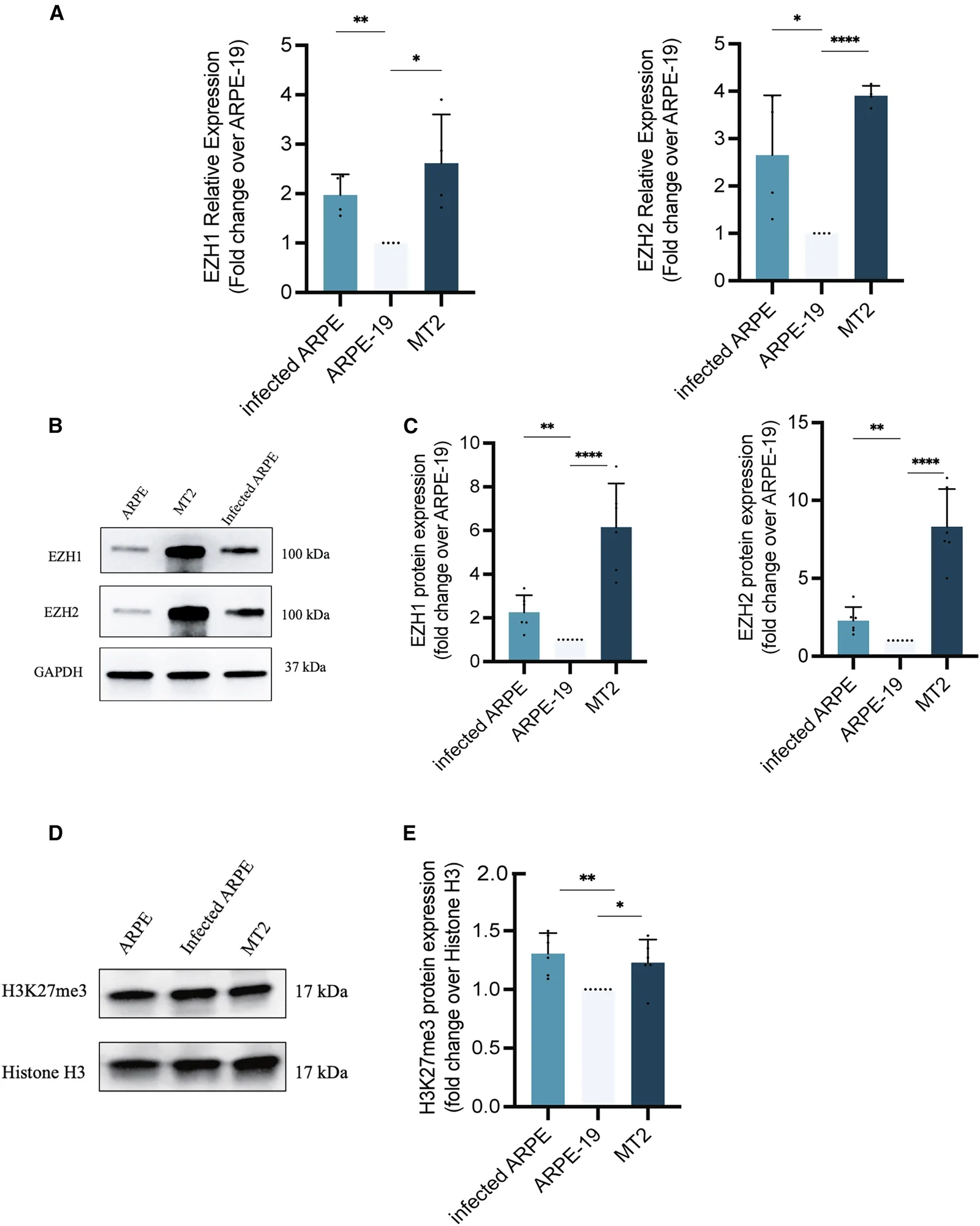
HTLV-1 infection is associated with increased EZH1, EZH2, and H3K27me3 levels in ARPE-19 cells EZH1, EZH2, and H3K27me3 expression levels were analyzed in ARPE-19 cells, HTLV-1-infected ARPE-19 cells, and MT-2 cells. GAPDH was used as the internal control for RT-qPCR and as the loading control for EZH1 and EZH2 western blotting. Histone H3 was used as the loading control for H3K27me3 western blotting. Data are shown as mean ± SD. Statistical significance was determined using one-way ANOVA with multiple comparisons. ∗*p* < 0.05, ∗∗*p* < 0.01, ∗∗∗*p* < 0.001, ∗∗∗∗*p* < 0.0001; ns, not significant. (A) EZH1 and EZH2 mRNA expression levels quantified by RT-qPCR. Expression levels were normalized to GAPDH and are shown relative to ARPE-19 cells (*n* = 4 per group). (B) Representative western blot images showing EZH1 and EZH2 protein expression in ARPE-19 cells, HTLV-1-infected ARPE-19 cells, and MT-2 cells. Original western blot images are provided in Data S1. (C) Quantification of EZH1 and EZH2 protein expression relative to GAPDH (*n* = 6 per group). (D) Representative western blot images showing H3K27me3 expression in ARPE-19 cells, HTLV-1-infected ARPE-19 cells, and MT-2 cells. Original western blot images are provided in Data S1. (E) Quantification of H3K27me3 expression relative to histone H3 (*n* = 6 per group).

H3K27me3 levels were significantly elevated in infected ARPE-19 (*p* < 0.01) and MT-2 cells (*p* < 0.05) (Figures 1D and 1E), suggesting that HTLV-1 infection induces a transcriptionally repressive chromatin state. This epigenetic shift may contribute to inflammatory activation and promote malignant progression. Together, these findings support the potential of valemetostat as a therapeutic strategy for HTLV-1-associated ocular inflammation and malignancy.

### Effects of valemetostat on ARPE-19 cell viability in HTLV-1-infected models

Preliminary dose- and time-response experiments used to determine the valemetostat concentrations and treatment duration are shown in Figure S1. To investigate the effects of valemetostat on ARPE-19 cell viability under HTLV-1 infection, two *in vitro* models were established. In the first model, irradiated MT-2 cells were co-cultured with ARPE-19 cells and treated with varying concentrations of valemetostat for 96 h. Following this, the co-cultured cells were passaged every two days until no MT-2 cells were microscopically detectable, ensuring the selective assessment of ARPE-19 cell viability post-treatment. In the second model, ARPE-19 cells were first infected through co-culture with irradiated MT-2 cells, and after the removal of MT-2 cells, the infected ARPE-19 cells were treated with valemetostat for 96 h. Co-cultures with TL-Om1 cells (HTLV-1 Tax-negative, less transcriptionally active) and Jurkat cells (HTLV-1-negative) served as controls.

ARPE-19 cell viability and viable cell enumeration were assessed (Figures 2A–2H). In the ARPE-MT-2 co-culture model, valemetostat reduced ARPE-19 viability at 1 μM (Figure 2A), accompanied by a corresponding decline in viable cell numbers (control: 103.07 ± 6.88, 0.01 μM: 102.53 ± 5.51, 0.1 μM: 99.93 ± 4.30, 1 μM: 87.93 ± 4.30) (Figure 2E). A similar but modest decrease was observed in directly infected ARPE-19 cells, with a significant reduction in viability at 1 μM (Figure 2B). The number of viable cells also decreased accordingly (control: 26.27 ± 1.58, 0.01 μM: 25.73 ± 5.12, 0.1 μM: 25.63 ± 2.65, 1 μM: 19.80 ± 3.90) (Figure 2F). In contrast, ARPE-19 cells co-cultured with TL-Om1 or Jurkat cells showed no significant viability changes across all concentrations (Figures 2C and 2D), and their viable cell counts remained stable (ARPE-19 + TL-Om1: control: 92.17 ± 6.93, 0.01 μM: 101.53 ± 8.62, 0.1 μM: 103.20 ± 4.95, 1 μM: 99.47 ± 3.19; ARPE-19 + Jurkat: control: 101.53 ± 7.99, 0.01 μM: 108.43 ± 11.08, 0.1 μM: 104.97 ± 13.16, 1 μM: 106.67 ± 4.73) (Figures 2G and 2H).

**Figure 2:**
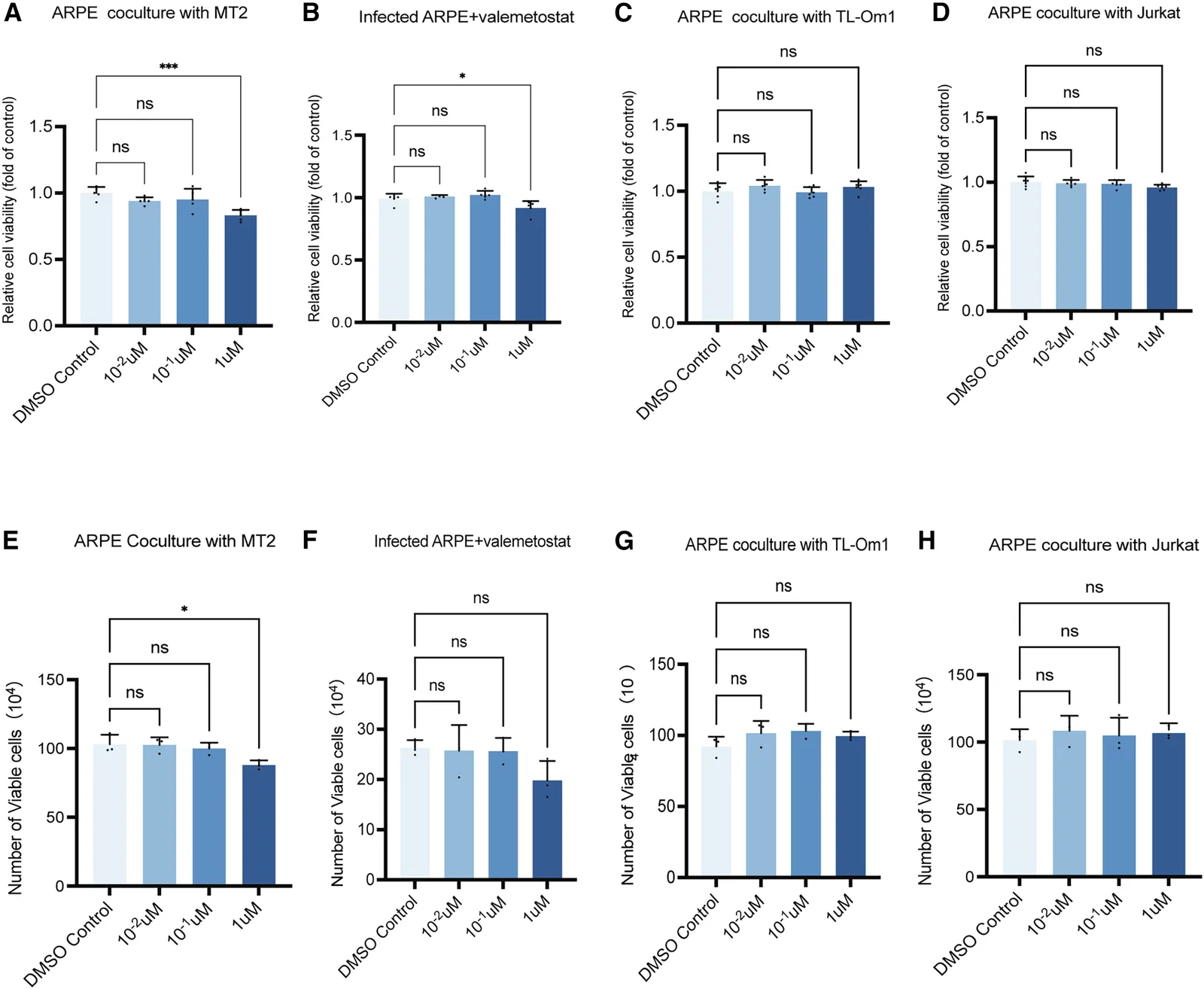
Effects of valemetostat on ARPE-19 cell viability in HTLV-1-related models ARPE-19 cell viability and viable cell number were assessed after treatment with valemetostat at 0.01, 0.1, or 1 μM for 96 h. DMSO-treated cells served as vehicle controls. Cell viability was measured using the CCK-8 assay (A–D), and viable cell number was determined by direct cell counting (E–H). Data are shown as mean ± SD from three independent biological replicates, and each dot represents one independent biological replicate. Statistical significance was determined using one-way ANOVA with multiple comparisons. ∗*p* < 0.05, ∗∗∗*p* < 0.001; ns, not significant. (A) ARPE-19 cells co-cultured with irradiated MT-2 cells (*n* = 3 per group). Valemetostat reduced ARPE-19 cell viability in the co-culture model. (B) Directly infected ARPE-19 cells (*n* = 3 per group). A mild reduction in cell viability was observed after treatment with 1 μM valemetostat, whereas lower concentrations showed no significant effect. (C) ARPE-19 cells co-cultured with irradiated TL-Om1 cells (*n* = 3 per group). No significant changes in cell viability were detected across valemetostat concentrations. (D) ARPE-19 cells co-cultured with irradiated Jurkat cells (*n* = 3 per group). No significant effects on ARPE-19 cell viability were observed. (E) ARPE-19 cells co-cultured with irradiated MT-2 cells (*n* = 3 per group). Treatment with 1 μM valemetostat reduced viable cell number. (F) Directly infected ARPE-19 cells (*n* = 3 per group). No significant changes in viable cell number were observed after valemetostat treatment. (G) ARPE-19 cells co-cultured with irradiated TL-Om1 cells (*n* = 3 per group). Valemetostat had no significant effect on viable cell number. (H) ARPE-19 cells co-cultured with irradiated Jurkat cells (*n* = 3 per group). No significant differences in viable cell number were observed across valemetostat concentrations.

These results indicate that valemetostat is associated with modest, context-dependent changes in ARPE-19 cell viability under HTLV-1-related conditions. The reduction observed in the ARPE-MT-2 co-culture model may reflect indirect effects related to the inflammatory and transcriptionally active environment generated by MT-2 cells. The modest decrease in viability observed in directly infected ARPE-19 cells suggests a measurable but limited effect under the current experimental conditions.

### Valemetostat exerts model-dependent effects on HTLV-1 proviral load and viral gene expression

To further evaluate the impact of valemetostat on HTLV-1 infection, we measured the proviral load (PVL) under different experimental conditions (Figure 3). Both the co-culture models and HTLV-1-infected ARPE-19 cells showed significantly higher PVL levels than control groups (*p* < 0.0001), confirming successful viral transmission and establishment of infection (Figures 3A and 3B). Drug analysis results showed that valemetostat treatment did not lead to a significant reduction in PVL in co-culture models using MT-2 and TL-Om1, nor in HTLV-1-infected ARPE-19 cells (Figures 3A–3C).

**Figure 3:**
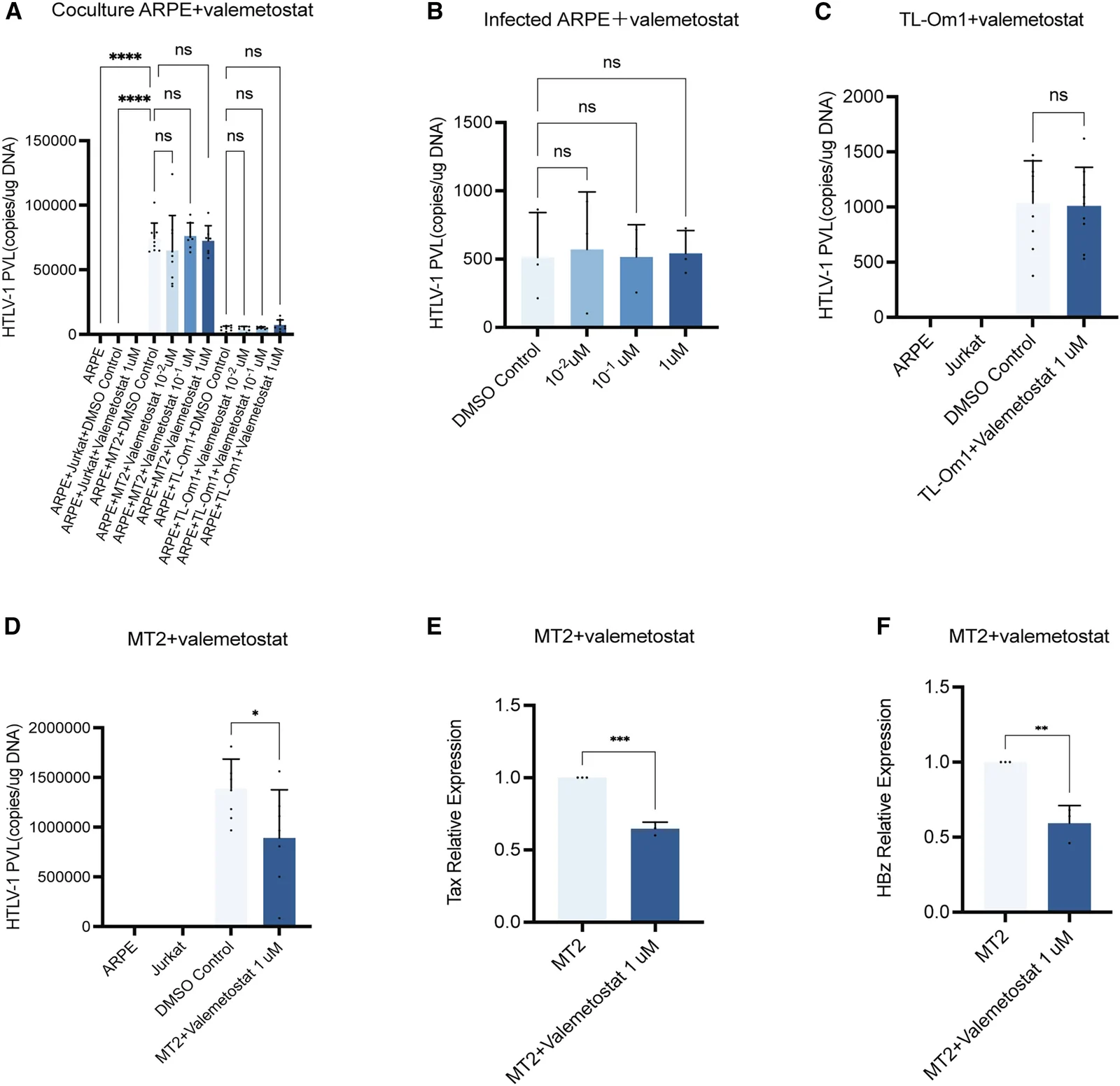
Valemetostat exerts model-dependent effects on HTLV-1 proviral load and viral gene expression HTLV-1 PVL and viral gene expression were analyzed in multiple cellular models following treatment with valemetostat. Cells were treated with valemetostat at the indicated concentrations for 96 h, and DMSO-treated cells served as vehicle controls. PVL was quantified by real-time quantitative PCR and expressed as copies per μg genomic DNA. Data are shown as mean ± SD from three independent biological replicates. Individual dots represent replicate measurements obtained from the independent experiments. Technical replicate measurements, when applicable, were averaged within each biological replicate before statistical analysis, and biological replicates were treated as the unit of analysis. Statistical significance was determined using one-way ANOVA with multiple comparisons or unpaired two-tailed Student’s *t* test, as appropriate. ∗*p* < 0.05, ∗∗*p* < 0.01, ∗∗∗*p* < 0.001, ∗∗∗∗*p* < 0.0001; ns, not significant. (A) ARPE-T-cell coculture model. ARPE-19 cells were cocultured with irradiated MT-2 or TL-Om1 cells and treated with valemetostat at 0.01, 0.1, or 1 μM (*n* = 3 per group). Coculture with HTLV-1-infected T cells increased PVL compared with uninfected ARPE-19 cells, whereas valemetostat did not significantly reduce PVL in this coculture setting. (B) Directly infected ARPE-19 cells. HTLV-1-infected ARPE-19 cells were treated with valemetostat at 0.01, 0.1, or 1 μM (*n* = 3 per group). No significant change in PVL was observed after valemetostat treatment. (C) TL-Om1 cells. TL-Om1 cells were treated with 1 μM valemetostat (*n* = 3 per group). PVL levels were not significantly different from those in DMSO-treated control cells. (D) MT-2 cells. MT-2 cells were treated with 1 μM valemetostat (*n* = 3 per group). Valemetostat significantly reduced HTLV-1 PVL in MT-2 cells (*n* = 3 per group). (E and F) Viral gene expression in MT-2 cells. Tax and HBZ mRNA expression levels were quantified by RT-qPCR in MT-2 cells following treatment with 1 μM valemetostat (*n* = 3 per group). Expression levels were normalized to GAPDH and are shown relative to DMSO-treated MT-2 cells.

In contrast, a significant reduction in PVL was observed specifically in MT-2 cells (Figure 3D), following treatment with 1 μM valemetostat (*p* < 0.05). To further assess viral transcriptional activity, we quantified the expression of the representative HTLV-1 transcripts Tax and HBZ. Consistent with the reduction in PVL, valemetostat treatment significantly suppressed the mRNA expression of both Tax (*p* < 0.001) and HBZ (*p* < 0.01) in MT-2 cells (Figures 3E and 3F).

Collectively, within the 4-day treatment period, this model-dependent virological effect of valemetostat may indirectly improve the HU microenvironment by primarily affecting HTLV-1 Tax-positive MT-2 cells.

#### Dose-dependent effects of valemetostat on cytokine and chemokine secretion

HTLV-1-infected CD4^+^ T cell clones derived from the eyes of patients with HU could secrete large amounts of inflammatory cytokines and chemokines, including interleukin (IL)-6, IL-8, and IL-10, all of which contribute to HU pathogenesis.^27^ To investigate the effects of valemetostat on inflammatory signaling in HU, we quantified IL-10, IL-12p70, CCL-5, CCL-2, and CXCL-10 using flow cytometry (Figure 4). IL-6, IL-8 (Figure 5), and NF-κB phosphorylation (Figure 6) were measured by ELISA.

**Figure 4:**
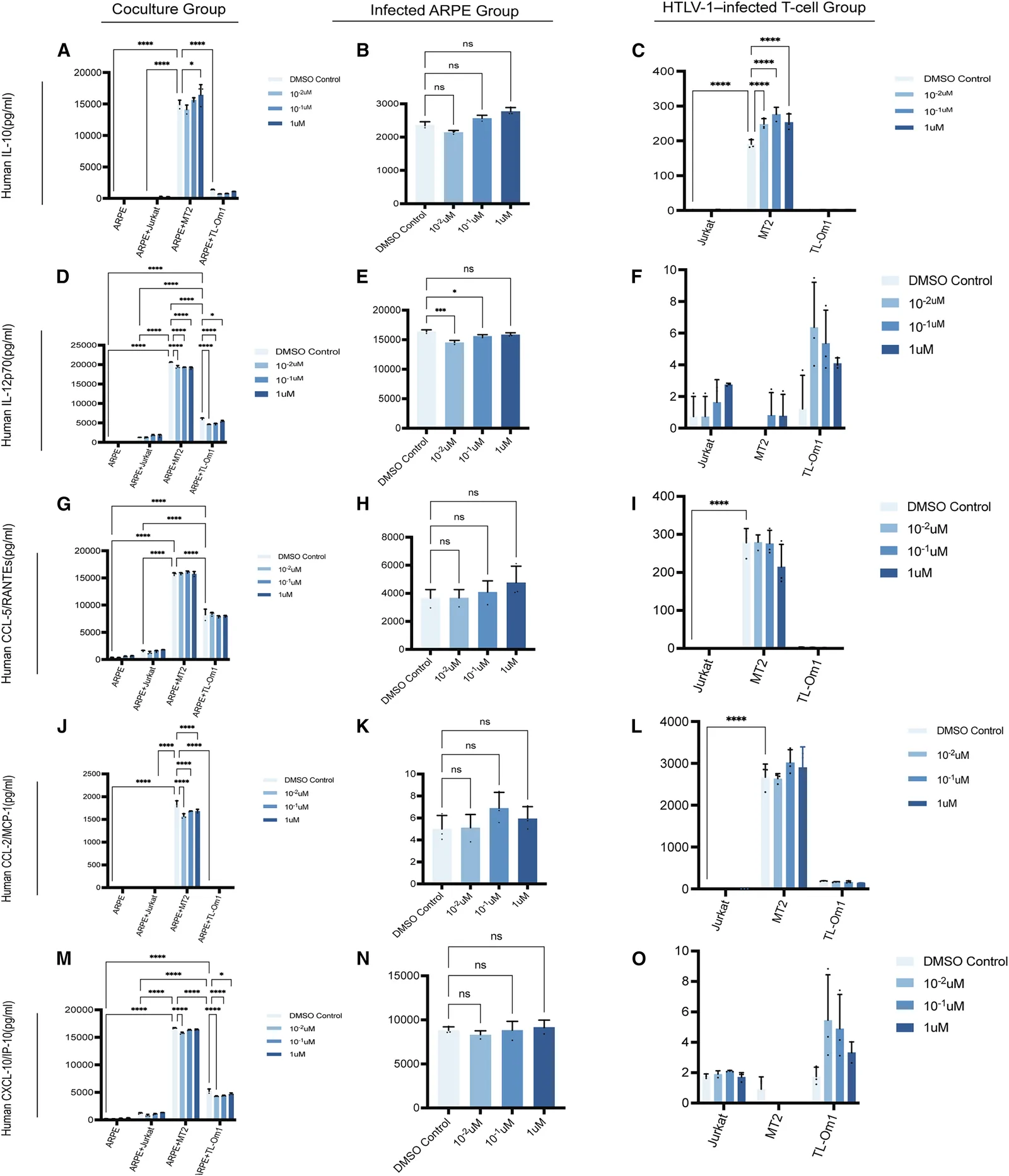
Valemetostat modulates cytokine and chemokine secretion in a model-dependent manner Cytokine and chemokine levels were quantified in culture supernatants from ARPE-T-cell co-culture models, directly infected ARPE-19 cells, and HTLV-1-infected T cells following treatment with DMSO or valemetostat at 0.01, 0.1, or 1 μM for 96 h. Cytokines and chemokines were measured using cytometric bead array and flow cytometry. Data are shown as mean ± SD from three independent biological replicates, and each dot represents one independent biological replicate. Statistical significance was determined using one-way ANOVA with multiple comparisons or the Kruskal-Wallis test, as appropriate. ∗*p* < 0.05, ∗∗*p* < 0.01, ∗∗∗*p* < 0.001, ∗∗∗∗*p* < 0.0001; ns, not significant. Detection limits were 1.9 pg/mL for IL-12p70, 3.3 pg/mL for IL-10, 3.7 pg/mL for TNF, 2.7 pg/mL for MCP-1/CCL2, 1.0 pg/mL for RANTES/CCL5, and 2.8 pg/mL for IP-10/CXCL10. (A–C) IL-10 levels in ARPE-T-cell co-culture models, directly infected ARPE-19 cells, and HTLV-1-infected T cells after valemetostat treatment (*n* = 3 per group). (D–F) IL-12p70 levels in ARPE-T-cell co-culture models, directly infected ARPE-19 cells, and HTLV-1-infected T cells after valemetostat treatment (*n* = 3 per group). (G–I) RANTES/CCL5 levels in ARPE-T-cell co-culture models, directly infected ARPE-19 cells, and HTLV-1-infected T cells after valemetostat treatment (*n* = 3 per group). (J–L) MCP-1/CCL2 levels in ARPE-T-cell co-culture models, directly infected ARPE-19 cells, and HTLV-1-infected T cells after valemetostat treatment (*n* = 3 per group). (M–O) IP-10/CXCL10 levels in ARPE-T-cell co-culture models, directly infected ARPE-19 cells, and HTLV-1-infected T cells after valemetostat treatment (*n* = 3 per group).

**Figure 5:**
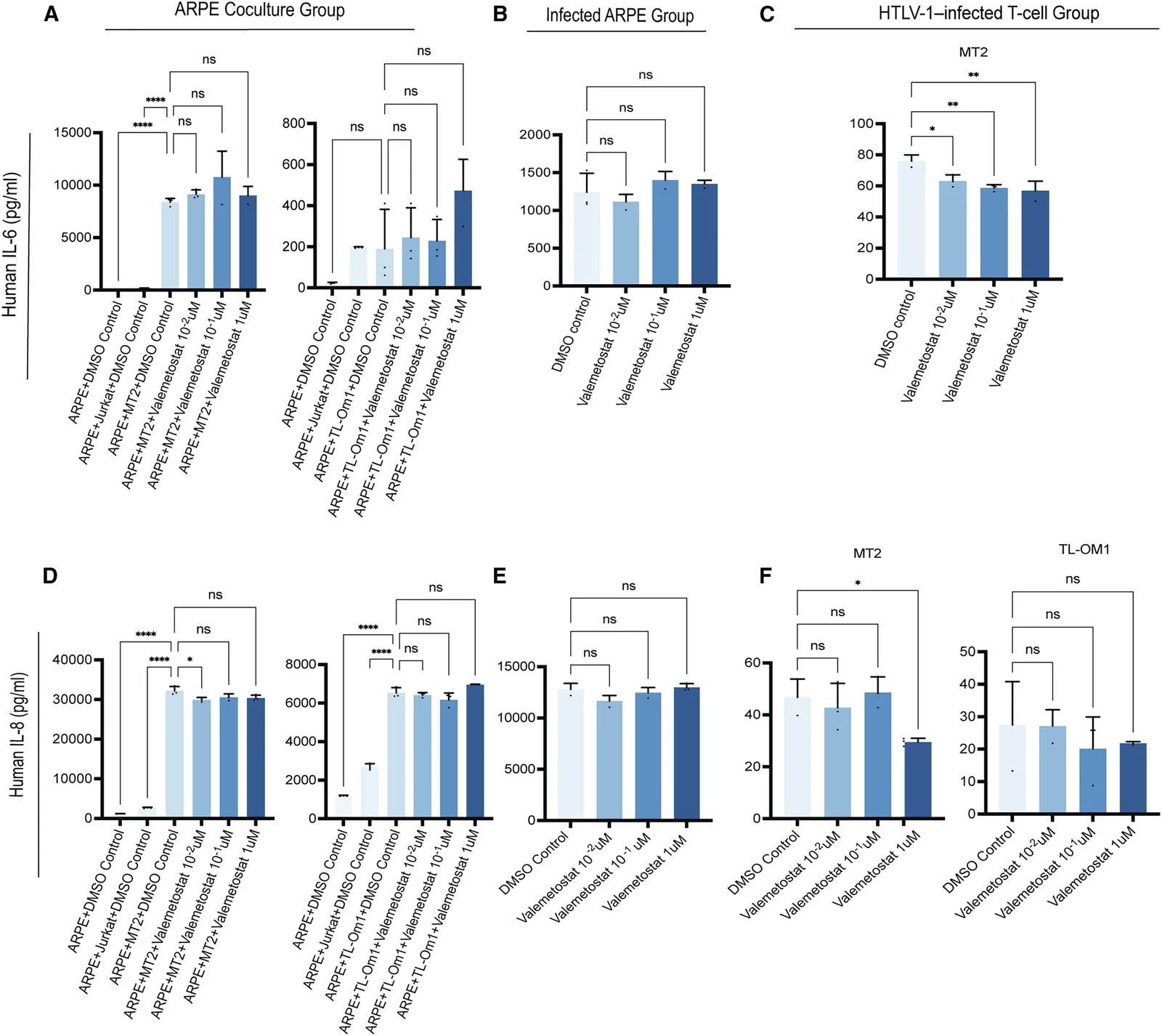
Valemetostat modulates IL-6 and IL-8 secretion in a model-dependent manner IL-6 and IL-8 levels were quantified in culture supernatants from ARPE-T-cell co-culture models, directly infected ARPE-19 cells, and HTLV-1-infected T cells after treatment with DMSO or valemetostat at 0.01, 0.1, or 1 μM for 96 h. IL-6 and IL-8 levels were measured by ELISA. Data are shown as mean ± SD from three independent biological replicates, and each dot represents one independent biological replicate. Statistical significance was determined using one-way ANOVA with multiple comparisons or the Kruskal-Wallis test, as appropriate. ∗*p* < 0.05, ∗∗*p* < 0.01, ∗∗∗*p* < 0.001, ∗∗∗∗*p* < 0.0001; ns, not significant. (A) IL-6 levels in ARPE-19 cells co-cultured with irradiated MT-2 or TL-Om1 cells following valemetostat treatment (*n* = 3 per group). (B) IL-6 levels in directly infected ARPE-19 cells following valemetostat treatment (*n* = 3 per group). (C) IL-6 levels in MT-2 cells following valemetostat treatment (*n* = 3 per group). (D) IL-8 levels in ARPE-19 cells co-cultured with irradiated MT-2 or TL-Om1 cells following valemetostat treatment (*n* = 3 per group). (E) IL-8 levels in directly infected ARPE-19 cells following valemetostat treatment (*n* = 3 per group). (F) IL-8 levels in MT-2 and TL-Om1 cells following valemetostat treatment (*n* = 3 per group).

**Figure 6:**
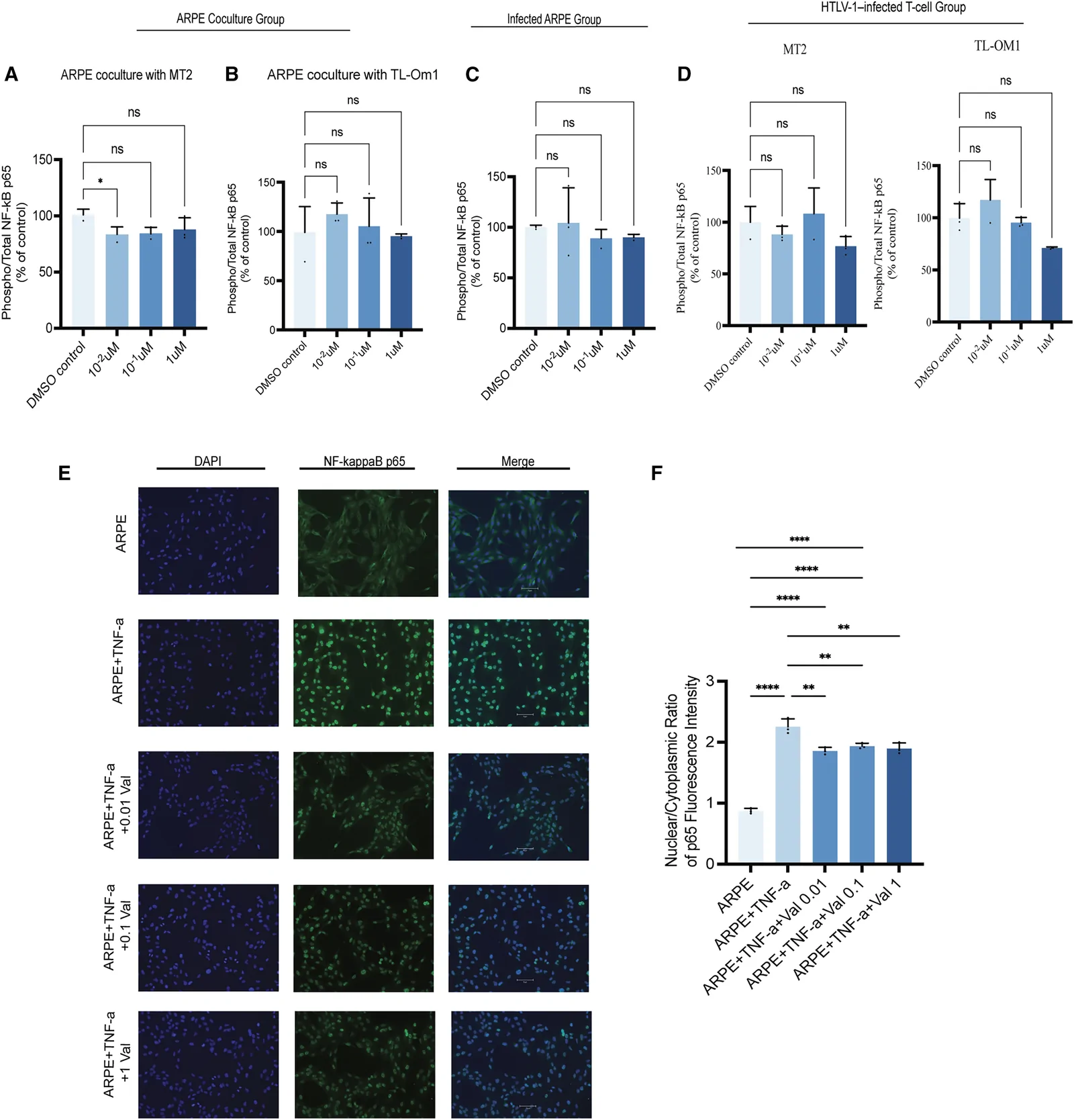
Valemetostat affects NF-κB p65 phosphorylation and nuclear localization in a context-dependent manner NF-κB p65 phosphorylation and nuclear localization were assessed in ARPE-T-cell co-culture models, directly infected ARPE-19 cells, HTLV-1-infected T cells, and TNF-α-stimulated ARPE-19 cells after valemetostat treatment. Phosphorylated NF-κB p65 levels were quantified relative to total NF-κB p65 and are shown as percentages relative to the corresponding DMSO-treated control (A–D). NF-κB p65 nuclear localization was assessed by immunofluorescence staining in ARPE-19 cells treated with valemetostat at 0.01, 0.1, or 1 μM for 24 h, followed by stimulation with TNF-α at 10 ng/mL for 30 min (E and F). Data are shown as mean ± SD from three independent biological replicates, and each dot represents one independent biological replicate. Statistical significance was determined using one-way ANOVA with multiple comparisons. ∗*p* < 0.05, ∗∗*p* < 0.01, ∗∗∗*p* < 0.001, ∗∗∗∗*p* < 0.0001; ns, not significant. (A) Phospho/total NF-κB p65 ratio in ARPE-19 cells co-cultured with irradiated MT-2 cells following valemetostat treatment (*n* = 3 per group). (B) Phospho/total NF-κB p65 ratio in ARPE-19 cells co-cultured with irradiated TL-Om1 cells following valemetostat treatment (*n* = 3 per group). (C) Phospho/total NF-κB p65 ratio in directly infected ARPE-19 cells following valemetostat treatment (*n* = 3 per group). (D) Phospho/total NF-κB p65 ratio in MT-2 and TL-Om1 cells following valemetostat treatment (*n* = 3 per group). (E) Representative immunofluorescence images of NF-κB p65 localization in ARPE-19 cells treated with valemetostat and stimulated with TNF-α. NF-κB p65 is shown in green, and nuclei were counterstained with DAPI in blue. Scale bars, 75 μm. (F) Quantification of the nuclear-to-cytoplasmic fluorescence intensity ratio of NF-κB p65 from immunofluorescence images (*n* = 3 per group). Nuclear and cytoplasmic fluorescence intensities were measured on a per-cell basis, and 30–50 cells were analyzed per condition in each experiment.

##### Anti-inflammatory cytokine IL-10

As shown in Figures 4A–4C, IL-10 secretion increased markedly in the ARPE-MT-2 co-culture model compared with untreated ARPE-19 cells and with Jurkat or TL-Om1 co-culture groups (*p* < 0.0001). IL-10 levels in the DMSO control were also elevated, indicating strong baseline activation induced by MT-2 co-culture.

Following valemetostat treatment, IL-10 secretion showed a modest but clear dose-dependent increase in both the ARPE-MT-2 co-culture system (Figure 4A) and MT-2 cells (Figure 4C). Significant elevation appeared at 1 μM in ARPE-MT-2 co-culture (*p* < 0.05) and at 0.01, 0.1, and 1 μM in MT-2 cells (*p* < 0.0001). In contrast, IL-10 levels remained unchanged in TL-Om1 cells and in directly HTLV-1-infected ARPE-19 cells across all concentrations (Figures 4A–4C).

##### Pro-inflammatory cytokine IL-12p70

IL-12p70 levels were significantly elevated in ARPE-19 cells co-cultured with MT-2 or TL-Om1 cells (*p* < 0.0001) (Figure 4D). Valemetostat reduced IL-12p70 expression at 0.01 μM and 0.1 μM (*p* < 0.0001) and at 1 μM (*p* < 0.05). A similar tendency was seen in directly infected ARPE-19 cells, with reductions at 0.01 μM (*p* < 0.001) and 0.1 μM (*p* < 0.05) but not at 1 μM (Figure 4E).

##### Interleukin-6

IL-6 secretion increased significantly in ARPE-MT-2 co-cultures compared with untreated ARPE-19 cells (*p* < 0.0001). However, valemetostat treatment did not significantly alter IL-6 levels in the MT-2 or TL-Om1 co-culture groups (Figure 5A). In HTLV-1-infected ARPE-19 cells, IL-6 secretion remained unchanged across all concentrations (Figure 5B). In MT-2 cells, IL-6 showed a dose-dependent reduction, with significant decreases at 0.01 μM (*p* < 0.05) and further decreases at 0.1 μM and 1 μM (*p* < 0.01) (Figure 5C).

##### Interleukin-8

IL-8 secretion was significantly elevated in ARPE-19 cells co-cultured with MT-2 or TL-Om1 cells (*p* < 0.0001). Low-dose valemetostat (0.01 μM) reduced IL-8 secretion in MT-2 co-culture (*p* < 0.05) (Figure 5D). IL-8 remained unchanged in infected ARPE-19 cells (Figure 5E). In HTLV-1-infected T-cells, valemetostat reduced IL-8 secretion only in MT-2 cells at 1 μM (*p* < 0.05), with no changes observed in TL-Om1 cells (Figure 5F).

##### Chemokine CCL-5 (RANTES)

CCL-5 levels were significantly elevated in ARPE-MT-2 and ARPE-TL-Om1 co-cultures (*p* < 0.0001) (Figure 4G). Valemetostat did not alter CCL-5 secretion in any model, including directly infected ARPE-19 cells (Figure 4H) or HTLV-1-infected T cell lines (Figure 4I).

##### CCL-2 (MCP-1)

CCL-2 expression increased markedly in ARPE-MT-2 co-cultures (*p* < 0.0001) and was reduced by valemetostat at 0.01 μM and 0.1 μM (*p* < 0.0001), but not at 1 μM (Figure 4J). Directly infected ARPE-19 cells showed no change with treatment (Figure 4K). HTLV-1-infected T cell lines displayed higher CCL-2 secretion in MT-2 cells than in TL-Om1 cells, without any drug-induced reduction (Figure 4L).

##### CXCL-10 (IP-10)

CXCL-10 levels were significantly elevated in ARPE-19 cells co-cultured with MT-2 and TL-Om1 cells. Valemetostat reduced CXCL-10 at 0.01 μM in MT-2 co-culture (*p* < 0.0001), and at 0.01 μM and 0.1 μM in TL-Om1 co-culture (*p* < 0.0001), with a smaller reduction at 1 μM (*p* < 0.05). No changes were observed in directly infected ARPE-19 cells (Figure 4N).

### Effects of valemetostat on NF-κB phosphorylation and nuclear localization

To investigate whether valemetostat affects NF-κB signaling in different HTLV-1-associated cellular contexts, we quantified NF-κB p65 phosphorylation under coculture, infection, and HTLV-1-infected T cell line conditions. In ARPE-MT-2 cocultures, a modest but statistically significant reduction in the phospho/total NF-κB p65 ratio was observed at 0.01 μM valemetostat compared with the DMSO control (*p* < 0.05), whereas no significant differences were detected at higher concentrations (Figure 6A). In ARPE-19 cells co-cultured with TL-Om1 cells, HTLV-1-infected ARPE-19 cells, and HTLV-1-infected T cell lines, NF-κB p65 phosphorylation levels remained unchanged across all treatment groups (Figures 6B–6D).

To further assess whether valemetostat influences the subcellular distribution of NF-κB p65, immunofluorescence staining was performed in ARPE-19 cells under TNF-α stimulation (Figures 6E and 6F). In TNF-α-treated ARPE-19 cells, NF-κB p65 exhibited increased nuclear localization compared with untreated controls (*p* < 0.0001). Valemetostat treatment did not abolish NF-κB nuclear localization; however, the nuclear-to-cytoplasmic p65 fluorescence intensity ratio was modestly reduced by valemetostat treatment (*p* < 0.01).

Our results show that valemetostat increases the level of the anti-inflammatory cytokine IL-10 in MT-2-containing conditions, including the ARPE-MT-2 co-culture system and MT-2 cells. In contrast, IL-10 remained unchanged in TL-Om1 co-culture and in directly HTLV-1-infected ARPE-19 cells. Pro-inflammatory cytokines and chemokines were modulated in a context-dependent manner. For example, IL-6 and IL-8 were reduced mainly in MT-2 cells and, to a lesser extent, in MT-2 co-culture at low dose. IL-12p70 and CXCL-10 were reduced in co-culture systems, whereas directly infected ARPE-19 cells showed limited changes. NF-κB signaling was only modestly attenuated, with a small reduction in phosphorylation in ARPE-MT-2 co-culture and preserved p65 nuclear localization under TNF-α stimulation.

These findings indicate model dependence and dose dependence after 96 h treatment. The improvement of the inflammatory microenvironment in HTLV-1-associated uveitis is mainly indirect. The drug acts primarily by modulating Tax-positive MT-2 cells, which show strong viral activity and exert dominant inflammatory effects.

### Valemetostat induces modest and model-dependent late apoptosis in ARPE-19 cells under HTLV-1-associated conditions

To evaluate the pro-apoptotic effects of valemetostat on ARPE-19 cells under different HTLV-1-related microenvironments, Annexin V-FITC/PI staining followed by flow cytometry was performed across four models (Figure 7). In the ARPE-Jurkat co-culture (Figure 7A) model, apoptosis remained unchanged. In the ARPE-TL-Om1 coculture model, valemetostat caused small but detectable increases in apoptosis: early apoptosis increased significantly at 1 μM (*p* < 0.01); late apoptosis showed a mild dose-dependent rise, becoming significant at 0.1 μM (*p* < 0.05) (Figure 7B). ARPE-19 cells co-cultured with MT-2 showed the clearest apoptotic response among the co-culture models: late apoptosis increased significantly at 0.1 μM (*p* < 0.05) and 1 μM (*p* < 0.01) (Figure 7C). Infected ARPE-19 cells showed modest but consistent apoptosis induction: early apoptosis increased at concentrations of 0.01 μM and 0.1 μM (*p* < 0.01 and *p* < 0.05, respectively). Late apoptosis trended upward with increasing concentration (*p* < 0.01) (Figure 7D).

**Figure 7:**
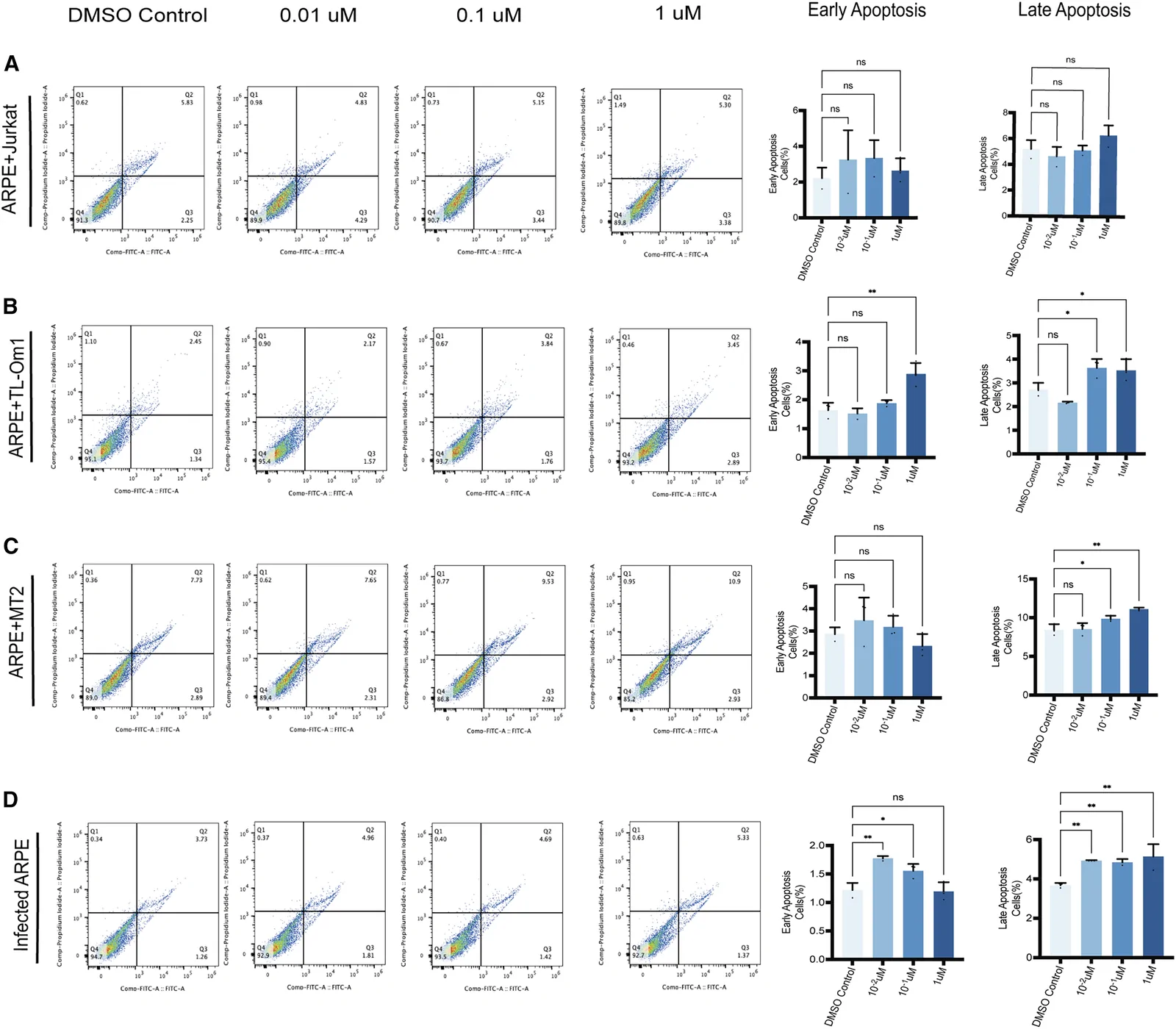
Valemetostat induces modest and model-dependent late apoptosis in ARPE-19 cells under HTLV-1-associated conditions Early and late apoptosis were assessed in ARPE-T-cell co-culture models and directly infected ARPE-19 cells after treatment with DMSO or valemetostat at 0.01, 0.1, or 1 μM for 96 h. Apoptosis was analyzed by Annexin V-FITC/PI staining and flow cytometry. Representative flow cytometry plots are shown on the left, and quantification is shown on the right. Data are shown as mean ± SD from three independent biological replicates, and each dot represents one independent biological replicate. Statistical significance was determined using one-way ANOVA with multiple comparisons or the Kruskal-Wallis test, as appropriate. ∗*p* < 0.05, ∗∗*p* < 0.01; ns, not significant. (A) Early and late apoptosis in ARPE-19 cells co-cultured with irradiated Jurkat cells following valemetostat treatment (*n* = 3 per group). (B) Early and late apoptosis in ARPE-19 cells co-cultured with irradiated TL-Om1 cells following valemetostat treatment (*n* = 3 per group). (C) Early and late apoptosis in ARPE-19 cells co-cultured with irradiated MT-2 cells following valemetostat treatment (*n* = 3 per group). (D) Early and late apoptosis in directly infected ARPE-19 cells following valemetostat treatment (*n* = 3 per group).

Together, these findings indicate that valemetostat is associated with modest, context-dependent pro-apoptotic effects, which are more evident in HTLV-1-related models, particularly in MT-2 co-culture and infected ARPE-19 systems. However, the overall magnitude of apoptosis remains limited (approximately 10%–15%) under the current experimental conditions. While these observations are reproducible across replicates, they suggest a measurable but modest biological response rather than a strong cytotoxic effect.

### Valemetostat lowers H3K27me3 in ARPE-19 cells under MT-2 exposure and HTLV-1 infection

We evaluated H3K27me3 levels using immunofluorescence staining in ARPE-19 cells under several coculture and infection conditions. Fluorescence intensity was measured as integrated density per DAPI-stained cell (Figure 8).

**Figure 8:**
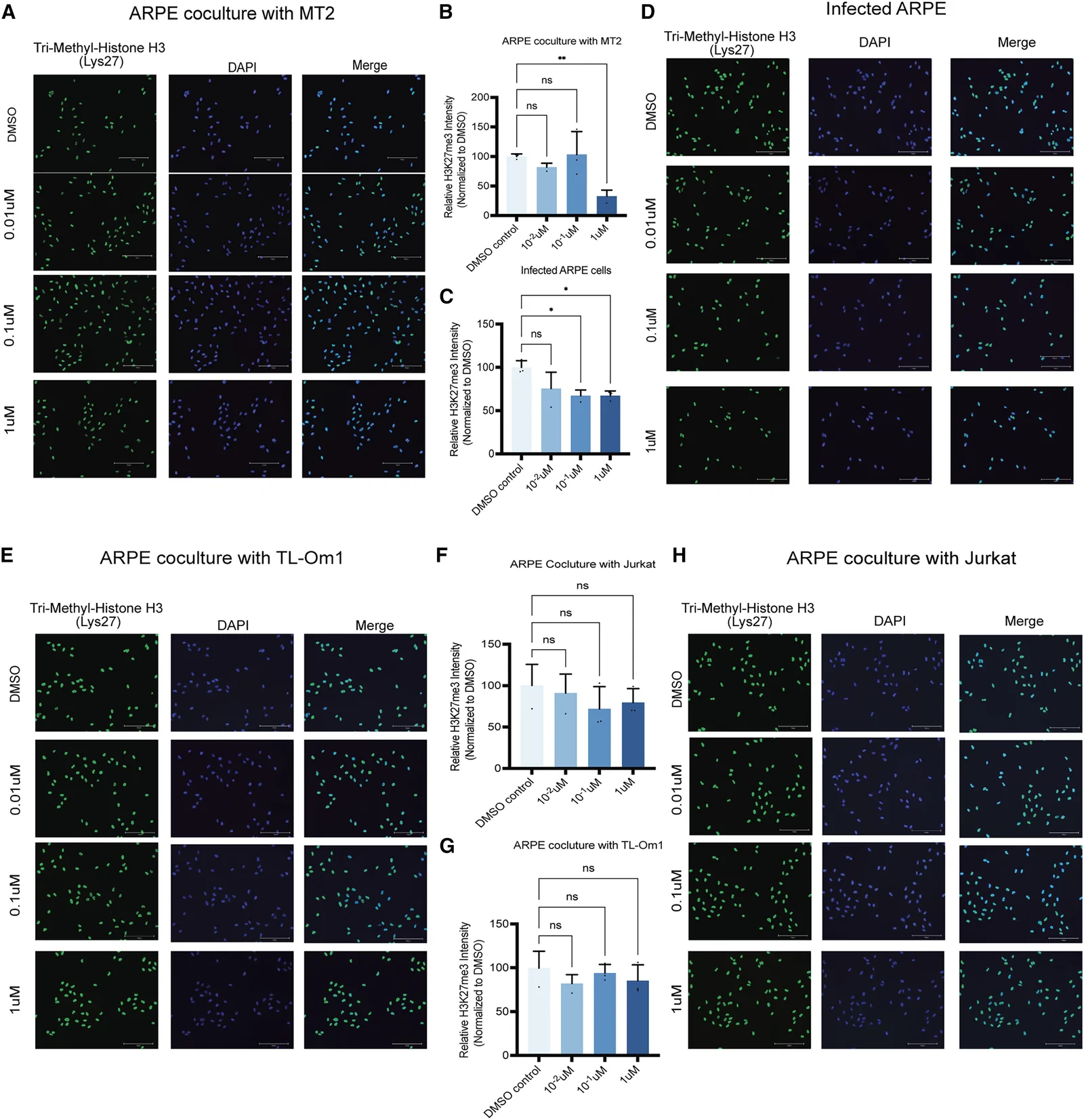
Valemetostat reduces H3K27me3 levels in ARPE-19 cells under HTLV-1-exposed conditions Representative immunofluorescence images and quantification of H3K27me3 expression in ARPE-19 cells under co-culture and direct infection models following treatment with valemetostat. Cells were treated with DMSO or valemetostat at the indicated concentrations for 96 h H3K27me3 is shown in green, and nuclei were counterstained with DAPI in blue. Fluorescence images were acquired at 20× magnification. Images were converted to 8-bit format for channel separation. H3K27me3 fluorescence intensity was quantified by dividing the integrated density by the total number of DAPI-stained cells and was normalized to the corresponding DMSO-treated control. Data are shown as mean ± SD from three independent biological replicates, and each dot represents one independent biological replicate. Statistical significance was determined by one-way ANOVA with multiple comparisons. ∗*p* < 0.05, ∗∗*p* < 0.01; ns, not significant. Scale bars, 150 μm. (A) Representative immunofluorescence images of ARPE-19 cells co-cultured with irradiated MT-2 cells and treated with DMSO or valemetostat at 0.01, 0.1, or 1 μM (*n* = 3 per group). (B) Quantification of H3K27me3 fluorescence intensity in ARPE-19 cells co-cultured with irradiated MT-2 cells (*n* = 3 per group). (C) Quantification of H3K27me3 fluorescence intensity in directly infected ARPE-19 cells generated by prior co-culture with irradiated MT-2 cells (*n* = 3 per group). (D) Representative immunofluorescence images of directly infected ARPE-19 cells treated with DMSO or valemetostat at 0.01, 0.1, or 1 μM (*n* = 3 per group). (E) Representative immunofluorescence images of ARPE-19 cells co-cultured with irradiated TL-Om1 cells and treated with DMSO or valemetostat at 0.01, 0.1, or 1 μM (*n* = 3 per group). (F) Quantification of H3K27me3 fluorescence intensity in ARPE-19 cells co-cultured with irradiated Jurkat cells (*n* = 3 per group). (G) Quantification of H3K27me3 fluorescence intensity in ARPE-19 cells co-cultured with irradiated TL-Om1 cells (*n* = 3 per group). (H) Representative immunofluorescence images of ARPE-19 cells co-cultured with irradiated Jurkat cells and treated with DMSO or valemetostat at 0.01, 0.1, or 1 μM (*n* = 3 per group).

In the MT-2 coculture model (Figure 8A), valemetostat produced a clear dose-dependent reduction in H3K27me3 associated fluorescence. Treatment with 1 μM showed a significant decrease compared with the DMSO control (*p* < 0.01) (Figure 8B). Infected ARPE-19 cells also showed reduced H3K27me3 expression. Significant decreases were observed at 0.1 μM and 1 μM (*p* < 0.05) (Figure 8C). Representative fluorescence images showed the corresponding reduction in H3K27me3 staining (Figure 8D). In contrast, ARPE-19 cells cocultured with TL-Om1 or Jurkat cells showed no significant changes in H3K27me3 at any valemetostat concentration (Figures 8E–8H).

Overall, these data demonstrate a model-dependent epigenetic response. Valemetostat reduces H3K27me3 in ARPE-19 cells in the presence of MT-2 cells or in HTLV-1-infected ARPE-19 cells. Suppression appears at 0.1 μM and becomes pronounced at 1 μM.

## Discussion

In this study, we characterized the multifaceted effects of valemetostat in HTLV-1-infected cells and highlighted its potential as a therapeutic agent for HU through epigenetic regulation, cytokine modulation, and effects on cellular viability. Taken together, our findings provide insight into the mechanism of action of valemetostat in HTLV-1-associated conditions, particularly HU, and suggest its potential as a therapeutic option.

### Epigenetic dysregulation in HU and the therapeutic potential of EZH inhibition

Epithelial tissues generally show low expression levels of EZH1 and EZH2.^28^ Here, we present the first evidence that HTLV-1 infection induces the upregulation of EZH1 and EZH2 expression as well as the repressive histone mark H3K27me3 in HTLV-1 infected ARPE-19 cells (Figure 1). These findings parallel those of Yamagishi et al., who reported that in ATL cells, EZH1 levels remain stable while EZH2 is markedly increased, accompanied by enhanced H3K27me3 deposition at promoter regions.^29^ Both EZH1 and EZH2 are required for ATL cell proliferation. Previous studies have demonstrated that EZH2 gain-of-function is a driver of oncogenesis in both solid tumors and hematologic malignancies,^30^^,^^31^ underscoring the importance of this epigenetic pathway in the context of HTLV-1. Notably, we found that treatment with valemetostat significantly reduced H3K27me3 levels in ARPE-19 cells co-cultured with MT-2 cells and in directly infected ARPE-19 cells (Figure 8). This suggests that valemetostat can counteract HTLV-1-induced epigenetic dysregulation. These results support the therapeutic potential of valemetostat in HU.

### Context-dependent impact of valemetostat on ARPE-19 cell viability in HTLV-1-related models

By assessing HTLV-1-infected T cell lines, co-culture systems, and HTLV-1-infected ARPE-19 models, we found that valemetostat treatment was associated with modest changes in ARPE-19 cell viability (Figure 2). In co-culture systems with highly infectious Tax-positive MT-2 cells and ARPE-19 cells directly infected with HTLV-1 by MT-2, valemetostat treatment resulted in a slight but statistically significant decrease in cell viability. No similar effect was observed in models with weak TL-Om1 infection capacity or in models co-cultured with HTLV-1-negative Jurkat cells. Apoptosis analysis showed similar results, with a modest increase in late-stage apoptosis in MT-2-related models (Figure 7). Consistent with these findings, Kurahashi et al. demonstrated that inhibiting DNA methylation and H3K27me3 accumulation effectively reprograms gene expression, suppressing tumor cell growth.^32^

It is noteworthy that although there were reproducible and significant differences compared to the DMSO control group, the absolute increase in late apoptosis was modest (typically, 1%–3% above baseline, depending on the model). Therefore, under the current experimental conditions, significant cytotoxic effects of the drug are not supported. Given the crucial role ARPE-19 cells play in maintaining retinal homeostasis, excessive impairment of ARPE-19 cell viability may be detrimental to eye health. Therefore, valemetostat was observed to have a stable but limited cytotoxicity effect within the experimental time frame.

### Differential effects of valemetostat on HTLV-1 transcription across cell models

Regarding viral parameters, valemetostat did not cause a significant change in HTLV-1 PVL in either the co-culture systems or HTLV-1-infected ARPE-19 cells. In contrast, treatment with 1 μM valemetostat reduced PVL in MT-2 cells. This reduction was accompanied by decreased mRNA expression of the HTLV-1-related genes Tax and HBZ, as measured by RT-qPCR (Figure 3). Distinct responses were observed among MT-2, TL-Om1, and Jurkat cells, highlighting the importance of cell line-specific differences in interpreting the effects of valemetostat. MT-2 cells exhibited strong infectivity, demonstrating active HTLV-1 transcription and strong Tax expression. In contrast, TL-Om1 and Jurkat cells showed weak or no infectivity.^33^

In conclusion, valemetostat’s effect on viral load and gene expression may vary depending on intracellular factors such as cell type, mode of infection, and transcriptional status. Within the time frame of our assays, the observed changes may reflect modulation of viral transcriptional activity and associated inflammatory signaling rather than direct antiviral or selective cytotoxic effects.

### Context-dependent effects of valemetostat on epigenetic remodeling and NF-κB-associated inflammatory signaling

Tax is known to activate the NF-κB pathway during the replicative phase of HTLV-1. Activated NF-κB enhances transcription from the viral long terminal repeat (LTR), thereby promoting viral replication.^34^ Because NF-κB plays a central role in both proviral transcription and inflammatory signaling, we examined whether valemetostat modulates NF-κB activity.

Our data show that valemetostat reduced H3K27me3 levels in both the MT-2 coculture system and infected ARPE-19 cells. However, the associated changes in cytokine production, apoptosis, and NF-κB activity were relatively modest. Several factors may explain this apparent discrepancy. First, NF-κB signaling and cytokine responses are not regulated by EZH1/2 alone. In our coculture models, HTLV-1 Tax constitutively activates upstream NF-κB signaling. Within the 96-h treatment window used in this study, EZH1/2 inhibition may be insufficient to counteract this strong Tax-driven activation. This may also explain why we did not observe a reversal of NF-κB nuclear translocation or a substantial induction of apoptosis, despite the substantial reduction in H3K27me3 levels.

Second, the magnitude of apoptotic and NF-κB responses depends on experimental conditions, including cell type, infection status, drug concentration, and duration of exposure. Under the conditions observed in this study, more pronounced effects were observed in MT-2 co-culture models characterized by higher HTLV-1 transcriptional activity. In this setting, valemetostat was associated with reductions in PVL and decreased expression of viral transcription markers (Tax and HBZ). In contrast, its effects on directly infected ARPE-19 cells were detectable but limited, which is consistent with the modest changes observed in cytokine secretion, cell viability, and NF-κB activity.

Together, these findings suggest that the reduction in H3K27me3 does not immediately translate into broad functional changes in inflammatory signaling in our study. Longer treatment durations and additional mechanistic studies will be required to determine whether epigenetic modulation can eventually alter NF-κB activity and HTLV-1-associated inflammatory signaling.

Taken together, our findings suggest that EZH1/2 inhibition induces measurable epigenetic changes that are accompanied by modest and context-dependent effects on viral transcription and inflammatory signaling. These results suggest that the primary impact of valemetostat in this system may relate to modulation of transcriptional and inflammatory responses.

### Valemetostat treatment upregulates IL-10 expression

EZH1/2 inhibition relieves H3K27me3-mediated transcriptional repression and can therefore reactivate silenced genes such as IL-10. Prior studies have shown that genetic loss of EZH2 increases IL-10 transcription in several CD4^+^ T cell subsets (Th0, Th2, and induced Treg cells), indicating direct PRC2 involvement in IL-10 regulation.^35^ Consistent with this mechanism, our results demonstrated a dose-dependent increase in IL-10 secretion across multiple HTLV-1-related models following valemetostat treatment, in agreement with previous reports using dual EZH1/2 inhibitors in HTLV-1-associated diseases.^24^

However, IL-10 plays context-dependent roles in HTLV-1 infection. Although it has anti-inflammatory functions, IL-10 can also promote the survival of HTLV-1-infected T cells and facilitate immune evasion.^7^ Clinically, patients with aggressive ATL often present with abnormally elevated IL-10 levels.^7^ In addition, IL-10 contributes to immune evasion by inhibiting the antigen-presenting function of dendritic cells and downregulating MHC class I molecules on tumor cells.^36^ Thus, IL-10 induction after EZH1/2 inhibition may reflect both a shift toward an anti-inflammatory milieu and a compensatory immunosuppressive response of HTLV-1-infected or transformed T cells.

Future strategies may benefit from combining EZH inhibition with IL-10 pathway blockade or other immunomodulatory approaches, as supported by evidence showing that IL-10 neutralization enhances ATL cell sensitivity to standard therapies and improves the efficacy of interferon-α/arsenic regimens.^37^

### Valemetostat response in the TL-Om1 ATL model

Compared with the highly infectious, Tax-positive MT-2 cells, the TL-Om1 line represents an ATL-derived HTLV-1-infected T cell clone and displays a disease biology distinct from the HU model. ATL is known to infiltrate ocular tissues and trigger intraocular inflammation.^38^^,^^39^^,^^40^ In our TL-Om1 model, valemetostat did not significantly reduce H3K27me3 enrichment, the PVL, or NF-κB activation within malignant T cells. Nevertheless, valemetostat markedly suppressed TL-Om1-induced CXCL10 secretion by ARPE-19 cells and induced apoptosis in a dose-dependent manner. These findings suggest that, in this model, the effects of valemetostat may be more closely related to modulation of the inflammatory microenvironment rather than direct cytotoxic effects on ATL cells.

In particular, the reduction of chemokine-driven leukocyte recruitment may help relieve ocular inflammation. By promoting the restoration of local immune homeostasis and supporting the resolution of chronic inflammatory responses, valemetostat may serve as a promising adjunctive therapy for ATL-related ocular disease.

### Limitations of the study

The scope of this study is constrained by the use of reductionist *in vitro* systems. Our models rely on co-cultures of HTLV-1-infected T cells with ARPE-19 cells and established HTLV-1-infected T cell lines, without performing infection transfer assays. While these approaches capture selected aspects of HTLV-1-associated inflammation and T cell malignancy, they do not fully recapitulate key features of the ocular microenvironment, including the blood-retinal barrier, retinal structure, or local immune regulation that define the ocular microenvironment. As a result, the *in vivo* behavior of valemetostat may require further validation.

The intraocular pharmacokinetics of valemetostat are also not defined. Systemic administration does not necessarily ensure sufficient drug penetration into posterior ocular tissues, and parameters such as bioavailability, distribution, and potential toxicity were not assessed in this study. Given that valemetostat is a clinically significant epigenetic drug with established systemic administration, understanding its ocular exposure and safety is crucial before considering its application in the treatment of HTLV-1-related uveitis.

Although the experimental design, replication, and statistical analyses were rigorously conducted, the broader biological and translational implications of these findings remain to be established. Future studies using *in vivo* systems or patient-derived models will be important to clarify whether the observed epigenetic and immunomodulatory effects of EZH1/2 inhibition can be translated into meaningful therapeutic outcomes in HTLV-1-associated ocular disease.

Because this study used established cell lines and commercially obtained primary human cells rather than prospectively enrolled human participants, sex was not incorporated as a biological variable in the experimental design, and sex-associated differences in the observed responses could not be evaluated. Gender was not applicable to these *in vitro* models. Future studies using donor-defined primary cells or patient-derived samples should evaluate whether biological sex influences the effects of EZH1/2 inhibition.

## Resource availability

### Lead contact

Further information and requests for resources and reagents should be directed to and will be fulfilled by the lead contact, Koju Kamoi (koju.oph@tmd.ac.jp).

### Materials availability

This study did not generate new unique reagents.

### Data and code availability

• All data supporting the findings of this study are available within the article and its supplemental information files.
• This study does not report original code.
• Any additional information required to reanalyze the data reported in this study is available from the lead contact upon request.

## Acknowledgments

This work was supported by 10.13039/501100001691JSPS 10.13039/501100001691KAKENHI (grant JP 20K09824, 25K02864), a Grant on Rare and Intractable Diseases from the Ministry of Health, Labour, and Welfare of Japan (grant 22FC0201), a Research Program on Emerging and Re-emerging Infectious Diseases grant from the 10.13039/100009619Japan Agency for Medical Research and Development, 10.13039/100009619AMED (grant 23fk0108671h0001, 23fk0108672h0001), a High-risk Emerging Infectious Diseases Research Grant from the 10.13039/100007449Takeda Science Foundation (FY2023) to K.K., and JST 10.13039/501100025019SPRING (grant no: JPMJSP2180) to Y. Zou.

## Author contributions

Conceptualization, K.K. and Y. Zou; methodology, K.K., Y. Zou, and M.Y.; software, Y. Zou; validation, K.K. and Y. Zou; formal analysis, K.K. and Y. Zou; investigation, K.K., Y. Zou, and M.Y.; resources, K.K. and Y. Zou; data curation, K.K. and Y. Zou; writing – original draft, Y. Zou; writing – review and editing, K.K., M.Y., Y. Zong, J.Z., and K.O.-M.; visualization, Y. Zou; supervision, K.K.; funding acquisition, K.K. and Y. Zou.

## Declaration of interests

The authors declare no competing interests.

## STAR★Methods

### Key resources table

**Table:** 

REAGENT or RESOURCE	SOURCE	IDENTIFIER
**Antibodies**		

Rabbit Monoclonal EZH1 antibody, 1:1000 dilution	Cell Signaling Technology	Cat# 42088; RRID: AB_2799212
Rabbit Monoclonal KMT6/EZH2 antibody, 1:1000 dilution	Abcam	Cat# ab191250; RRID: AB_2933983
Rabbit Monoclonal H3K27me3 antibody, 1:1000 dilution	ThermoFisher Scientific	Cat# MA5-11198; RRID: AB_11000749.
Rabbit Polyclonal Histone H3 antibody, 1:2000 dilution	Abcam	Cat# ab1791; RRID: AB_302613
Mouse Monoclonal GAPDH antibody, 1:2000 dilution	MBL	Cat# M171-3; RRID: AB_10597731
Secondary antibody: Sheep anti-mouse IgG HRP-linked secondary antibody, 1:5000 dilution	GE Healthcare (UK)	Cat# NA931; RRID: AB_772210
Secondary antibody: Donkey anti-rabbit IgG HRP-linked secondary antibody, 1:5000 dilution	GE Healthcare (UK)	Cat# NA934; RRID: AB_772206
Rabbit monoclonal NF-κB p65 (D14E12) antibody, 1:500 dilution	Cell Signaling Technology	Cat# 8242; RRID: AB_10859369
Goat anti-rabbit IgG H&L Alexa Fluor 488 secondary antibody, 1:2000 dilution	Abcam	Cat# ab150085

**Chemicals, peptides, and recombinant proteins**		

DMEM/F-12	Thermo Fisher Scientific (Gibco)	Cat# 11320033
Fetal Bovine Serum (Heat-inactivated)	Biowest	Cat# S1810
Penicillin–Streptomycin (10,000 U/mL)	Thermo Fisher Scientific	Cat# 15140-122
Trabecular Meshwork Cell Medium	ScienCell Research Laboratories	Cat# 6591
Fetal Bovine Serum (for TM medium)	GE Healthcare Japan	Cat# SH30070.03
Fibroblast Growth Supplement	ScienCell Research Laboratories	Cat# 2352
RPMI-1640	Wako Pure Chemical Corporation	Cat# 187-02705
EZH1/2 dual inhibitor (Valemetostat/DS-3201)	Selleck Chemicals, Yokohama, Japan	Cat# S8926
DMSO	Wako	Cat# 041-29351
Phosphate-Buffered Saline (PBS)	Thermo Fisher Scientific	Cat# 10010023
Paraformaldehyde, 4%	Leagene Biotechnology	Cat# DF0135
Triton™ X-100	Thermo Fisher Scientific	Cat# 28314
Bovine Serum Albumin (BSA)	Sigma-Aldrich	Cat# A9418
TNF-α	R&D Systems	Cat# 210-TA-005
DAPI-Fluoromount-G	Thermo Fisher Scientific	Cat# 00-4959-52
Tween 20	Thermo Fisher Scientific	Cat# BP337-500
RIPA buffer	Thermo Fisher Scientific	Cat# 89900
Protease Inhibitor Cocktail	Thermo Fisher Scientific	Cat# 87786
4–15% SDS-PAGE gel	Bio-Rad	Cat# 4561084
PVDF membrane	Millipore	Cat# IPVH00010
Bullet Blocking One	Nacalai Tesque	Cat# 13779-01
ECL solution	Bio-Rad	Cat# 1705060
RNase-free water	Thermo Fisher Scientific	Cat# AM9930
TBST	Takara	Cat# T972
10× Tris/Glycine/SDS	Bio-Rad	Cat# 1610732
10× Tris/Glycine	Bio-Rad	Cat# 1610734
10× PBS	Bio-Rad	Cat# 1610780
Ethanol 99.5%	Wako	Cat# 056-00485
Ponceau S Staining Solution	Thermo Fisher Scientific	Cat# A40000279
2-Mercaptoethanol	Wako	Cat# 13575-45
Laemmli buffer	Bio-Rad	Cat# 1610737
Normal Goat Serum	Thermo Fisher Scientific	Cat# 31872
TE buffer (PH = 8.0)	FUJIFILM	Cat# 314-90021

**Critical commercial assays**		

Annexin V-FITC/PI (propidium iodide) staining kit	Abcam	Cat# ab14085
CBA Human Inflammatory Cytokine kit	BD, Biosciences	Cat# 551811; RRID: AB_2868941
CBA Human Chemokine kit	BD, Biosciences	Cat# 552990; RRID: AB_2868970
Human IL-8 ELISA Kit	Thermo Fisher Scientific	Cat# KAC1301
Human IL-6 ELISA Kit	Thermo Fisher Scientific	Cat# EH2IL6
EZ1 Virus Mini Kit v2.0	QIAGEN	Cat# 955134
CCK-8 assay	AbMole Bioscience	Cat# M4839
NF-κB InstantOne ELISA Kit	eBioscience	Cat# 85-86083-11
Protein concentration detection: BCA assay kit	Thermo Fisher Scientific	Cat# 23227
RNeasy Kits for RNA Purification	Qiagen	Cat# 74104
SuperScript™ III Platinum™ SYBR™ Green One-Step qRT-PCR Kit	Thermo Fisher Scientific	Cat# 11736051

**Experimental models: Cell lines**		

Human cell line: ARPE-19	ATCC	Cat# CRL-2302; RRID: CVCL_0145
Primary human cells: hTMCs	ScienCell Research Laboratories	Cat# 6590
Human cell line: MT-2	JCRB Cell Bank (Osaka, Japan)	JCRB1210; RRID: CVCL_2631
Human cell line: TL-Om1	The Institute of Medical Science, The University of Tokyo	RRID: CVCL_B473
Human cell line: Jurkat	ATCC	Cat# TIB-152; RRID: CVCL_0367

**Oligonucleotides**		

RT-qPCR primers: EZH1-F	This study; synthesized by NIHON TECHNO SERVICE CO., LTD	5′-CAATTCAAGCTGGCGAAGAG-3′
RT-qPCR primers: EZH1-R	This study; synthesized by NIHON TECHNO SERVICE CO., LTD	5′-CAAGACAGTGCCGCTACCA-3′
RT-qPCR primers: EZH2-F	This study; synthesized by NIHON TECHNO SERVICE CO., LTD	5′-GCCAAGAGAGCCATCCAGAC-3′
RT-qPCR primers: EZH2-R	This study; synthesized by NIHON TECHNO SERVICE CO., LTD	5′-CCGACATACTTCAGGGCATCA-3′
RT-qPCR primers: Tax-F	This study; synthesized by NIHON TECHNO SERVICE CO., LTD	5′- TCAACCCTCACCACTCCAGGC-3′
RT-qPCR primers: Tax-R	This study; synthesized by NIHON TECHNO SERVICE CO., LTD	5′-GGGGCTCATGGTCATTGTCAT-3′
RT-qPCR primers: HBZ-F	This study; synthesized by NIHON TECHNO SERVICE CO., LTD	5′-AAGAGCATCGACGACATCGG-3′
RT-qPCR primers: HBZ-R	This study; synthesized by NIHON TECHNO SERVICE CO., LTD	5′- GTGGGACAGGAGCTTGAAGT-3′
Proviral load primers: HTLV-1 Tax-F	This study; synthesized by NIHON TECHNO SERVICE CO., LTD	5′-CCCACTTCCCAGGGTTTGGA-3′
Proviral load primers: HTLV-1 Tax-R	This study; synthesized by NIHON TECHNO SERVICE CO., LTD	5′-GGCCAGTAGGGCGTGA-3′
Proviral load primers: probe	This study; synthesized by NIHON TECHNO SERVICE CO., LTD	5′-FAM-CCAGTCTACGTGTTTG GAGACTGTGTACA-TAMRA-3′
GAPDH-F	This study; synthesized by NIHON TECHNO SERVICE CO., LTD	5′-GGTCTCCTCCGACTTCAACA-3′
GAPDH-R	This study; synthesized by NIHON TECHNO SERVICE CO., LTD	5′-TGCTGTAGCCAAATTCGTTG-3′

**Software and algorithms**		

Microplate reader	Power Wave XS; BIO-TEK, USA	https://www.marshallscientific.com/
Countess 3 Automated Cell Counter	Thermo Fisher Scientific	https://www.thermofisher.com/jp/
BD Analysis Software	BD Biosciences	https://www.bdbiosciences.com/en-us/products/software
NanoDrop 1000 system	Thermo Fisher Scientific	https://www.thermofisher.com/jp/
LightCycler 480 system	Roche	https://diagnostics.roche.com/jp/ja/products/instruments/lightcycler-480-ins-445.html
BD FACSLyric™ flow cytometer	BD Biosciences	https://www.bdbiosciences.com/ja-jp/products/instruments/flow-cytometers/clinical-cell-analyzers/facslyric
FlowJo software version 10.8.1	FlowJo, LLC	https://www.flowjo.com/docs/flowjo10/
Inverted fluorescence microscope EVOS M5000	Thermo Fisher Scientific	https://www.thermofisher.com/jp/
ImageJ software (version 1.54)	National Institutes of Health, USA	https://wsr.imagej.net/
Graphpad Prism version 6.02	GraphPad Software, CA, USA	https://www.graphpad.com/
ChemiDoc MP system	Bio-Rad	https://www.bio-rad.com/ja-jp/
Adobe Illustrator 2025	Adobe	https://www.adobe.com/ca/
MBR-1520R-4 X-ray generator	Hitachi Medical Corporation, Japan	https://www.hitachi-power-solutions.com/

### Experimental model and study participant details

#### Cell lines and culture conditions

ARPE-19 cells (CRL-2302) were obtained from the American Type Culture Collection (ATCC) and cultured in DMEM: F12 medium supplemented with 10% heat-inactivated fetal bovine serum and 1% penicillin-streptomycin solution. ARPE-19 cells used in this study were between passages 3 and 18. Human trabecular meshwork cells (hTMCs) were purchased from ScienCell Research Laboratories. hTMCs were isolated from the juxtacanalicular and corneoscleral regions of the human eye and were used between passages 10 and 20. hTMCs were cultured in trabecular meshwork cell medium supplemented with 2% fetal bovine serum, 1% fibroblast growth supplement, and 1% penicillin-streptomycin.

MT-2 cells were obtained from the JCRB Cell Bank. TL-Om1 cells were provided by the Institute of Medical Science, The University of Tokyo. MT-2 cells are HTLV-1 Tax-positive cells and represent an actively replicating viral phenotype. TL-Om1 cells are Tax-negative cells and represent a transcriptionally latent HTLV-1 phenotype. Jurkat cells (TIB-152) were obtained from ATCC and used as an HTLV-1-negative T cell control. MT-2, TL-Om1, and Jurkat cells were cultured in RPMI-1640 medium supplemented with 10% heat-inactivated fetal bovine serum and 1% penicillin-streptomycin.

Cell lines and primary cells were obtained from recognized cell banks, commercial suppliers, or academic sources as indicated in the key resources table. Available source-provided identity, characterization, and quality-control information was used where available. ARPE-19 and Jurkat cells were supplied by ATCC with STR profiling information and were reported by ATCC to be negative for mycoplasma contamination. hTMCs were characterized by ScienCell Research Laboratories by immunofluorescence using markers including alpha-smooth muscle actin and/or fibronectin and were reported by the supplier to be negative for mycoplasma, bacteria, yeast, and fungi. MT-2 cells were reported by JCRB to be negative for mycoplasma contamination. No additional independent authentication or mycoplasma testing was performed in our laboratory after receipt of the cells. Cells were maintained and used within the indicated passage ranges. All cells were maintained at 37°C in a humidified atmosphere containing 5% CO2, and cells used for assays were in the exponential growth phase.

### Method details

#### HTLV-1 infection model

HTLV-1 infection by co-culture was performed according to standard methods. ARPE-19 cells were seeded into six-well plates at 5 × 10ˆ4 cells per well and allowed to attach overnight. MT-2, TL-Om1, and Jurkat cells were irradiated with 9000 rads using an MBR-1520R-4 X-ray generator to stop proliferation while preserving cell-cell interaction capacity. Irradiated T cells were centrifuged, resuspended in fresh medium, and added to ARPE-19 cells at 1.5 × 10ˆ5 cells per well, corresponding to an ARPE-19: T cell ratio of 1:3.

Three co-culture systems were established: ARPE-19 + MT-2, used to model the inflammatory environment characteristic of HTLV-1 uveitis; ARPE-19 + TL-Om1, used as a model of ATL-related ocular infiltration with largely latent HTLV-1 transcription; and ARPE-19 + Jurkat, used as an HTLV-1-negative control.

Co-cultures were maintained for 96 h to allow viral transfer or cellular interaction. Residual irradiated T cells were removed every other day by passaging, during which adherent ARPE-19 cells were washed, trypsinized, and replated. After no T cells were microscopically detectable, these ARPE-19 cells were defined as directly infected ARPE-19 cells and used for downstream assays, including drug treatment.

#### Treatment with EZH1/2 dual inhibitor valemetostat

An EZH1/2 dual inhibitor (valemetostat, DS-3201) was dissolved in DMSO to a 10 mM stock and stored at −80 °C. Working concentrations (0.01, 0.1, 1 μM) were prepared fresh in culture medium immediately before use. To determine the appropriate drug concentration and treatment duration of valemetostat in the HU model, we conducted a preliminary experiment. Using the co-culture system, we first ensured that the drug exhibited no cytotoxicity toward normal ocular cells. Specifically, 5×10^4^ ARPE-19 cells were seeded per well of a six-well plate, and after adhesion, they were treated with different concentrations of valemetostat. The number of ARPE-19 cells was assessed on days 4, 6, and 8, revealing that valemetostat treatment had no significant impact on cell proliferation.

Next, to determine the optimal treatment duration, MT-2 cells were treated with valemetostat at concentrations ranging from 0.1 nM to 10,000 nM for 48, 72, and 96 h followed by cell viability assessment using the Cell Counting Kit-8 (CCK-8) assay. A concentration-dependent decrease in MT-2 cell viability was observed after 96 h, beginning at 10 nM. Subsequently, ARPE-19 cells and hTMCs were subjected to a CCK-8 cytotoxicity assay 96 h after treatment with different concentrations of the drug. The results demonstrated that both cell types exhibited a decline in viability only after treatment with 10 μM of the drug. We selected 0.01 μM, 0.1 μM, and 1 μM as the three treatment concentrations based on these findings. These concentrations did not affect ocular cells but effectively induced cytotoxicity in HTLV-1-infected T cell lines. DMSO was used as the vehicle control (Figures S1A–S1G). All the reagents are listed in key resources table.

#### Cell viability assay

The effect of valemetostat treatment on ARPE-19, hTMC, MT-2, and TL-Om1 cell viability was assessed using a CCK-8 assay (AbMole Bioscience). For adherent cells, ARPE-19 and hTMCs treated with valemetostat (1.5×10^4^ cells/well) were cultured in a 96-well plate for 96 h, followed by incubation with the CCK-8 reagent, which was dissolved in fresh culture medium, for 2 h. Similarly, TL-Om1 and MT-2 cells (8×10^3^ cells/well) were seeded in 96-well plates and treated with valemetostat for 96 h, followed by incubation with CCK-8 reagent dissolved in fresh medium for 2–3 h. Absorbance was measured at 450 nm using a microplate reader (Power Wave XS; BIO-TEK, USA). The experiments were carried out in three independent replicates. All the reagents are listed in key resources table.

#### Cell counting

Cell counting was performed using a Countess 3 Automated Cell Counter according to the manufacturer’s instructions. ARPE-19 cells were initially seeded at 5×10^4^ cells per well. To avoid interference from T cell counting, co-cultured cells were counted four days after valemetostat treatment, whereas infected cells were counted immediately after treatment completion. A 10 μL aliquot of uniformly mixed cell suspension was loaded into a cell counting plate. Experiments were performed in three independent replicates.

#### NF-κB phosphorylation ELISA

The level of NF-κB phosphorylation in infected ARPE-19 cells was quantified using an InstantOne ELISA Kit (eBioscience) following the manufacturer’s instructions. Absorbance at 450 nm was measured using a microplate reader. The experiments were carried out in three independent replicates. Key resources table outlines the reagents that were used.

#### Cytokine and chemokine measurement

Inflammatory cytokines and chemokines were quantified using the CBA Human Inflammatory Cytokine Kit and Chemokine Kit (BD Biosciences), as well as IL-6 and IL-8 ELISA Kits, following the manufacturer’s protocols. Briefly, ARPE-19 cells (5×10^4^ cells/well) were co-cultured with irradiated Jurkat, MT-2, or TL-Om1 cells (1.5×10^5^ cells/well) in 12-well plates for 96 h. The culture supernatants were then centrifuged (1000rpm, 5min), collected, and stored at −80°C until analysis. For cytokine and chemokine detection, 50 μL of the culture supernatant was incubated with a mixture of capture beads and phycoerythrin (PE)-conjugated detection reagent for 3 h, washed, and analyzed using BD Analysis Software (BD Biosciences). Standard curves were generated using the standards provided in each kit. The cytokines measured included IL-12p70, IL-10, TNF, and IL-6, while the chemokines measured included IL-8, MCP-1, RANTES, and IP-10. The detection limits were 1.9 pg/mL for IL-12p70, 3.3 pg/mL for IL-10, 3.7 pg/mL for TNF, 2.7 pg/mL for MCP-1, 1.0 pg/mL for RANTES, and 2.8 pg/mL for IP-10. All the experiments were carried out in three independent replicates. Key resources table outlines the reagents that were used.

#### HTLV-1 proviral load measurement

Genomic DNA was extracted from ARPE-19 cells using the EZ1 Virus Mini Kit v2.0, and DNA concentration was measured using a NanoDrop 1000 system. HTLV-1 proviral load was quantified by real-time PCR using a Roche 480 system. Glyceraldehyde-3-phosphate dehydrogenase (GAPDH) was used as the internal control. Each experiment was performed in three independent biological replicates, with each sample analyzed in technical triplicate. Primer and probe sequences are listed in the key resources table.

#### Annexin V-FITC/PI apoptosis assay

ARPE-19 cells co-cultured with X-ray-irradiated MT-2, TL-Om1, or Jurkat cells, as well as directly infected ARPE-19 cells after complete removal of MT-2 cells, were treated with 0, 0.01, 0.1, or 1 μM valemetostat. Cells were collected, and apoptosis was assessed using Annexin V-FITC/PI staining (Abcam). Apoptotic cells were analyzed using a BD FACSLyric flow cytometer, and data were processed using FlowJo software version 10.8.1 (BD Biosciences). Experiments were performed in three independent replicates.

#### Immunofluorescence staining and image analysis

ARPE-19 cells were seeded in 24-well plates at a density of 3×10^4^ cells per well and cultured overnight. The following day, the spent culture medium was removed, and the cells were fixed with 4% paraformaldehyde for 15 min at room temperature (20°C–25 °C). Permeabilization was performed using 0.1% Triton X-100 for 15 min. Subsequently, the cells were blocked with 1% bovine serum albumin (BSA) for 1 h at room temperature and incubated overnight at 4°C with primary antibodies diluted in antibody dilution buffer.

For histone modification analysis, cells were incubated with an anti-H3K27me3 antibody diluted in 0.1% BSA (Thermo Fisher Scientific). For NF-κB functional analysis, cells were stimulated with recombinant human TNF-α (10 ng/mL) for 30 min as a positive control and then incubated with an anti-NF-κB p65 antibody (CST) diluted in 1% BSA and 0.3% Triton X-100.

The next day, cells were washed three times with 0.1% PBST and incubated with goat anti-rabbit IgG Alexa Fluor 488-conjugated secondary antibodies (Abcam) at room temperature in the dark for 1 h. Following three additional washes with 0.1% PBST, the nuclei were stained with DAPI (4′,6-diamidino-2-phenylindole)-Fluoromount-G for 5 min at 37°C in the dark. Fluorescence images were acquired using an inverted fluorescence microscope (EVOS M5000, Thermo Fisher Scientific) at 20× magnification. Fluorescence intensity measurements were performed using ImageJ software (version 1.54). Images were converted to 8-bit format for channel separation, and fluorescence intensity was quantified by dividing the integrated density by the total number of DAPI-stained cells. To evaluate the subcellular distribution of NF-κB p65, the nuclear-to-cytoplasmic (N/C) fluorescence intensity ratio was quantified using immunofluorescence microscopy. Three independent experiments were conducted for each group. Nuclear regions were defined by DAPI staining, and cytoplasmic regions were determined by excluding the nuclear area. Mean fluorescence intensities of NF-κB p65 in the nucleus and cytoplasm were measured, and the N/C ratio was calculated on a per-cell basis. Key resources table outlines the reagents and antibodies that were employed, respectively.

#### Western blotting

Cells were cultured in 6-well plates until a confluent monolayer was formed. Proteins were extracted using RIPA buffer supplemented with a protease inhibitor cocktail. Lysates were centrifuged at 14,000 rpm for 20 min at 4°C. Protein concentration was determined using a BCA assay kit. For western blot analysis, 5–20 μg of protein was separated on a 4–15% SDS-PAGE gel and transferred onto polyvinylidene difluoride membranes. The membranes were blocked with Bullet Blocking One buffer for 5 min at room temperature, followed by overnight incubation at 4°C with the following primary antibodies: anti-EZH1 (CST), anti-EZH2 (Abcam), anti-H3K27me3 (Thermo Fisher Scientific), anti-Histone H3 (Abcam), and anti-GAPDH (MBL). After five TBST washes, the membranes were incubated for 1 h with either anti-mouse IgG peroxidase (GE Healthcare) or anti-rabbit IgG peroxidase (GE Healthcare) at room temperature. Signals were detected using an ECL solution and imaged with a ChemiDoc MP system. Band intensities were quantified using ImageJ software. Key resources table outlines the reagents and antibodies that were employed, respectively. These experiments were performed in independent triplicate.

#### RNA isolation and quantitative real-time RT-PCR

Total RNA was isolated using a Qiashredder (Qiagen) and purified using an RNeasy Mini Kit (Qiagen), following the manufacturer’s specifications. The yield and purity of the RNA were measured using a NanoDrop 1000 system (Thermo Fisher Scientific). RNA was then subjected to quantitative real-time PCR (RT-PCR) using the SuperScript III Platinum SYBR Green One-Step qRT-PCR Kit (Thermo Fisher Scientific), according to the manufacturer’s instructions. A conventional PCR was initially performed to obtain an amplification product suitable for the construction of standard curves using RT-PCR procedures. The incorporation of SYBR Green into the PCR products was monitored in real-time after each PCR cycle, enabling the calculation of the threshold cycle (Ct) value, which defines the PCR cycle number at which an exponential number of PCR products is produced. The thermocycling conditions were as follows: 3 min at 50°C, 5 min at 95°C, and 40 cycles of 15 s at 95°C, 30 s at 60°C. All RT-qPCR experiments were performed using independent biological replicates, with each sample analyzed in technical triplicate. The relative expression was calculated using the 2−ΔΔCt method. The exact number of biological replicates for each experiment is provided in the corresponding figure legend.

### Quantification and statistical analysis

Data are presented as mean ± standard deviation. All *in vitro* experiments were performed with at least three independent biological replicates unless otherwise stated. Statistical analyses were performed using GraphPad Prism 6.02. Group differences were assessed using Student’s *t* test, the Kruskal-Wallis test, or one-way ANOVA, as appropriate. All tests were two-sided, and *p* < 0.05 was considered statistically significant. Asterisks indicate statistical significance as follows: ∗*p* < 0.05, ∗∗*p* < 0.01, ∗∗∗*p* < 0.001, and ∗∗∗∗*p* < 0.0001.

### Additional resources

This study did not generate additional resources.
