# Supplementary material for: Dual impact of EZH1/2-targeted therapy in HTLV-1-associated inflammation: Direct and indirect mechanisms
Source: iScience. 2026 Oct 9;29(11):117775. doi: 10.1016/j.isci.2026.117775 (PMC13677313; doi:10.1016/j.isci.2026.117775)
Supplement: Supplementary file 1 [file mmc1.pdf]

iScience, Volume *29*

Supplemental information

Dual impact of EZH1/2-targeted therapy in HTLV-1-associated inflammation: Direct and indirect mechanisms

Yaru Zou, Koju Kamoi, Mingming Yang, Yuan Zong, Jing Zhang, and Kyoko Ohno-Matsui

Supplemental information

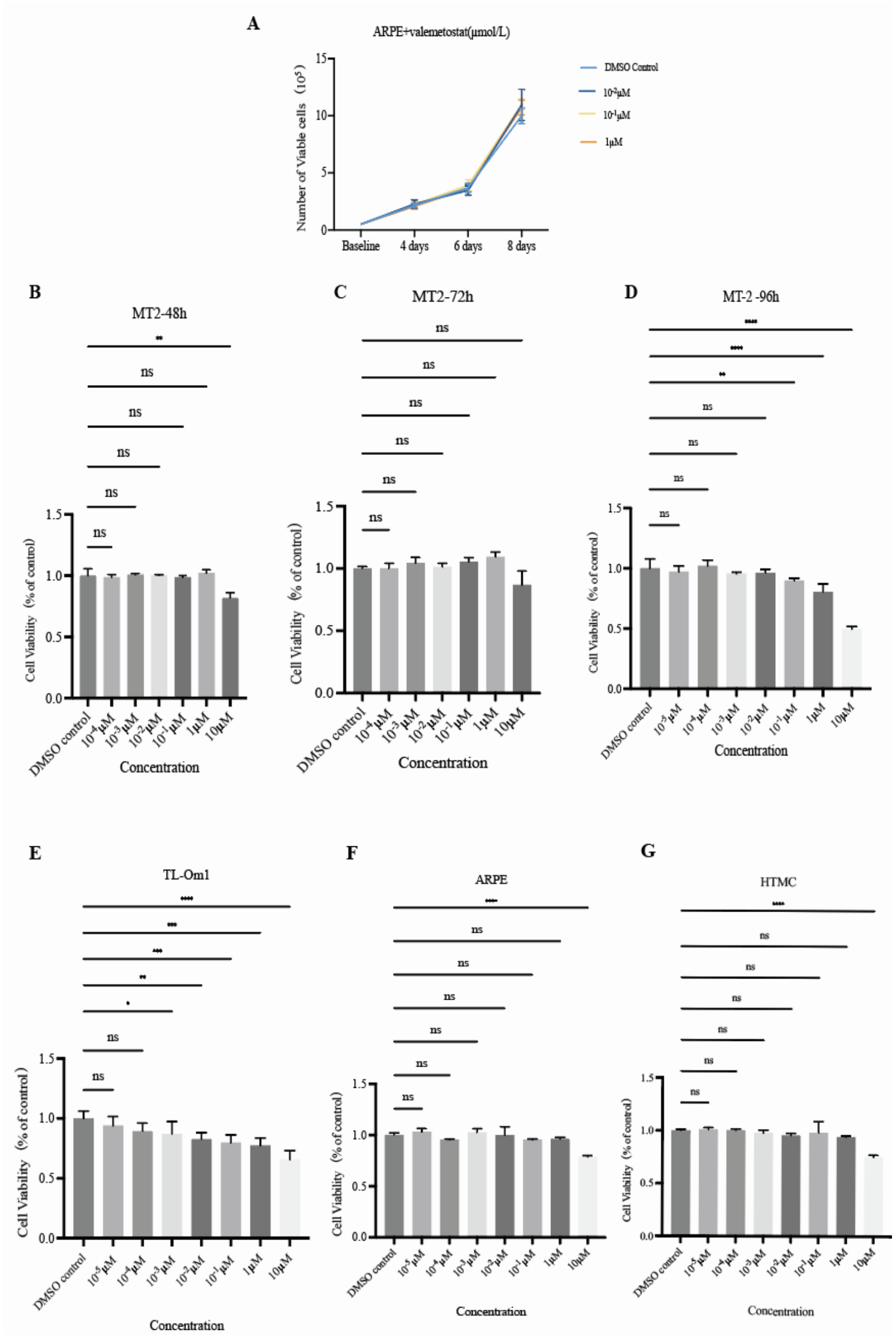

Figure S1. Effects of valemetostat on cell proliferation and viability in ARPE-19 cells, HTLV-1-infected T-cell lines, and control cells.
(A) ARPE-19 cells were treated with valemetostat at concentrations of 10^{-4} μM, 10^{-3} μM, 10^{-2} μM, and 1 μM, with 0.1% DMSO as vehicle control. Cell proliferation was assessed by direct cell counting at baseline, 4, 6, and 8 days.
(B-D) MT2 cells were treated with valemetostat (10^{-4} μM to 10 μM) for 48 h (B), 72 h (C), and 96 h (D), and cell viability was determined by CCK-8 assay.
(E-G) TL-Om1 cells (E), ARPE-19 cells (F), and human trabecular meshwork cells (HTMC) (G) were treated with valemetostat (10^{-5} μM to 10 μM) for 96 h, and cell viability was measured using CCK-8 assay. For the CCK-8 assay, 10 μL of CCK-8 reagent was added to each well and incubated for 2 h at 37°C, and absorbance was measured at 450 nm.
All experiments were performed in triplicate. Data represent mean ± SD. Statistical analysis was performed using one-way ANOVA. *$p < 0.05$, **$p < 0.01$, ***$p < 0.001$; ns: not significant.

Data S1: Original uncropped western blot images used in Figure 1.

Figure 1B

whole membrane

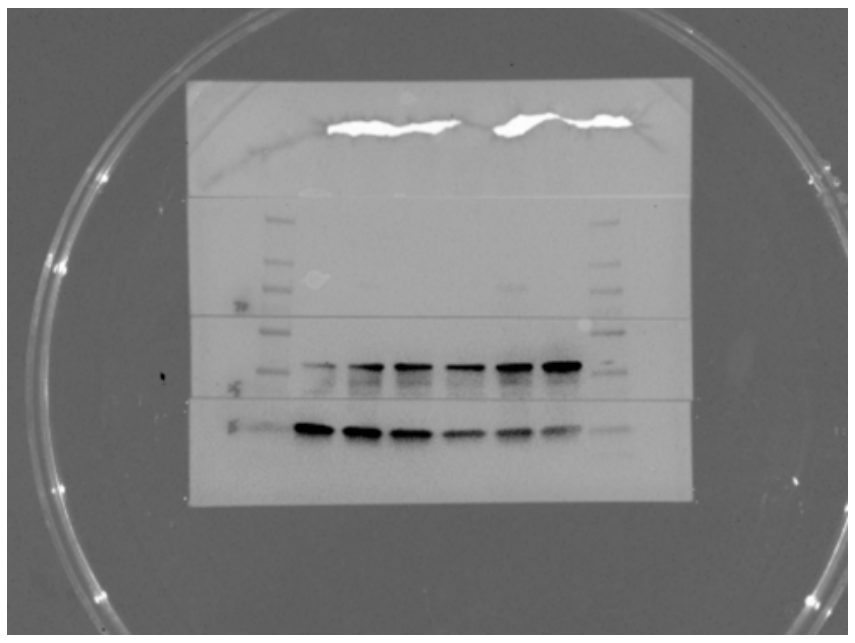

GAPDH

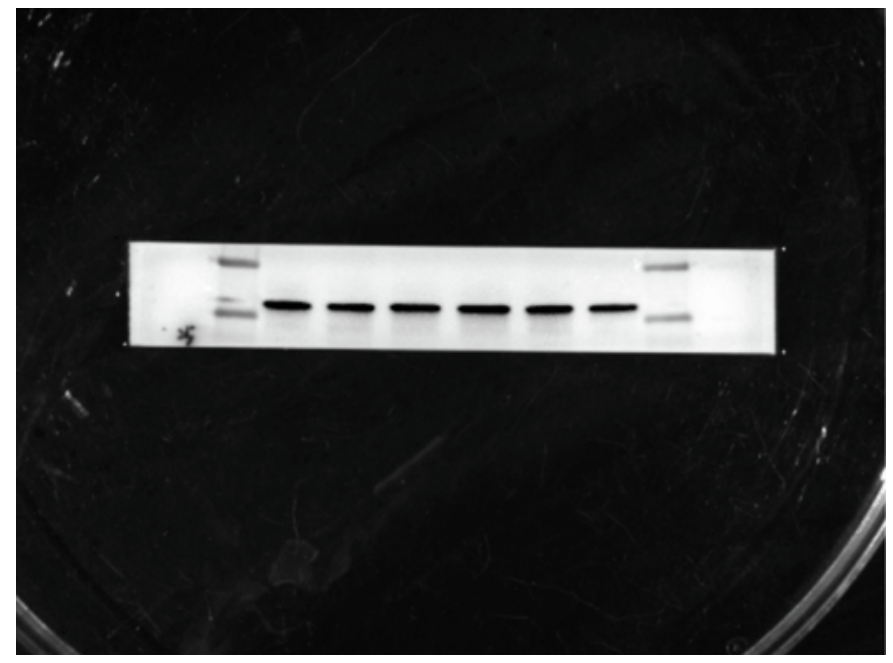

EZH1

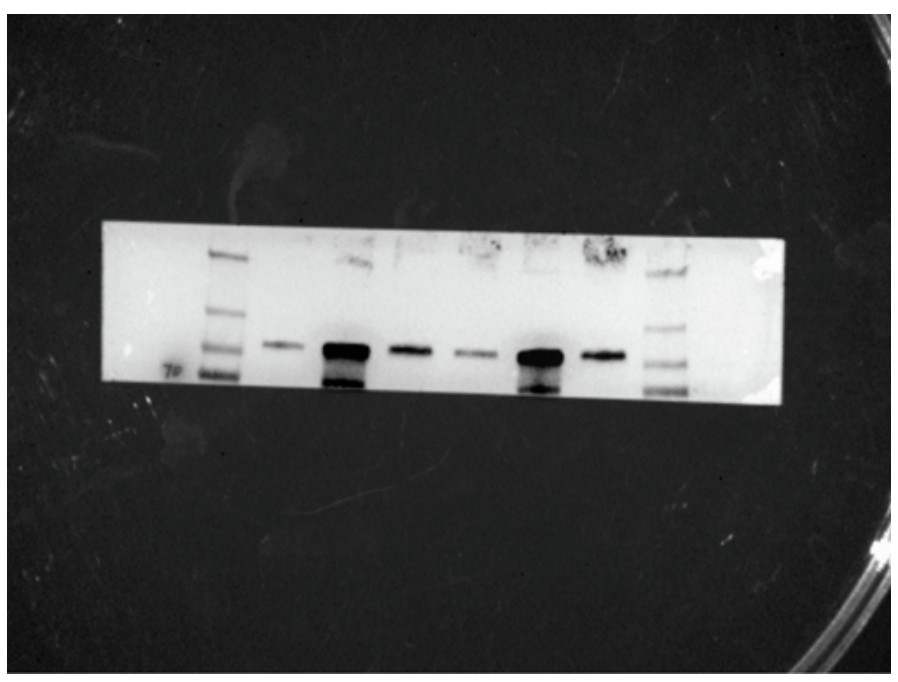

EZH2

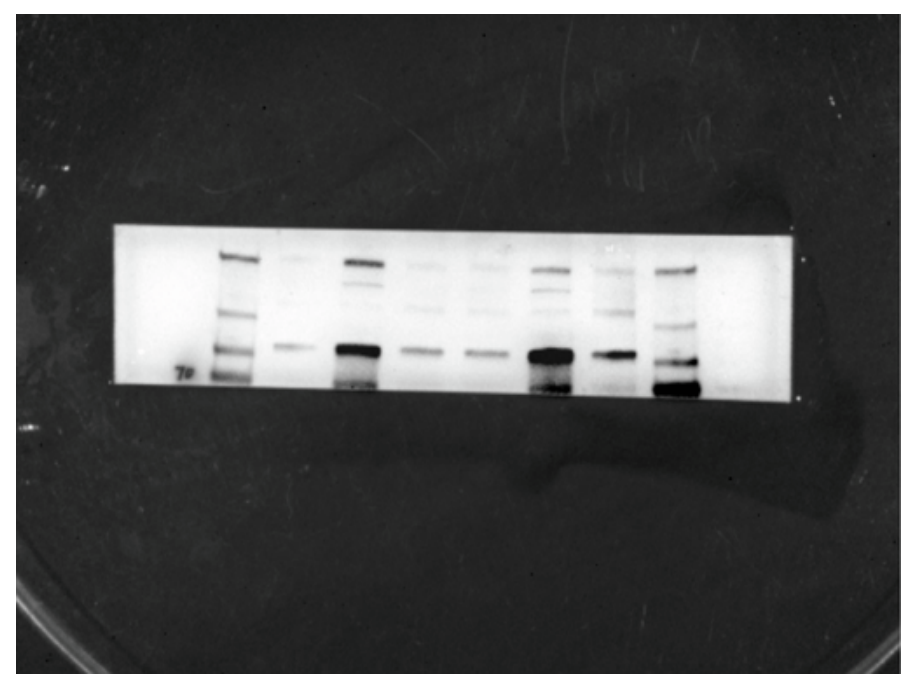

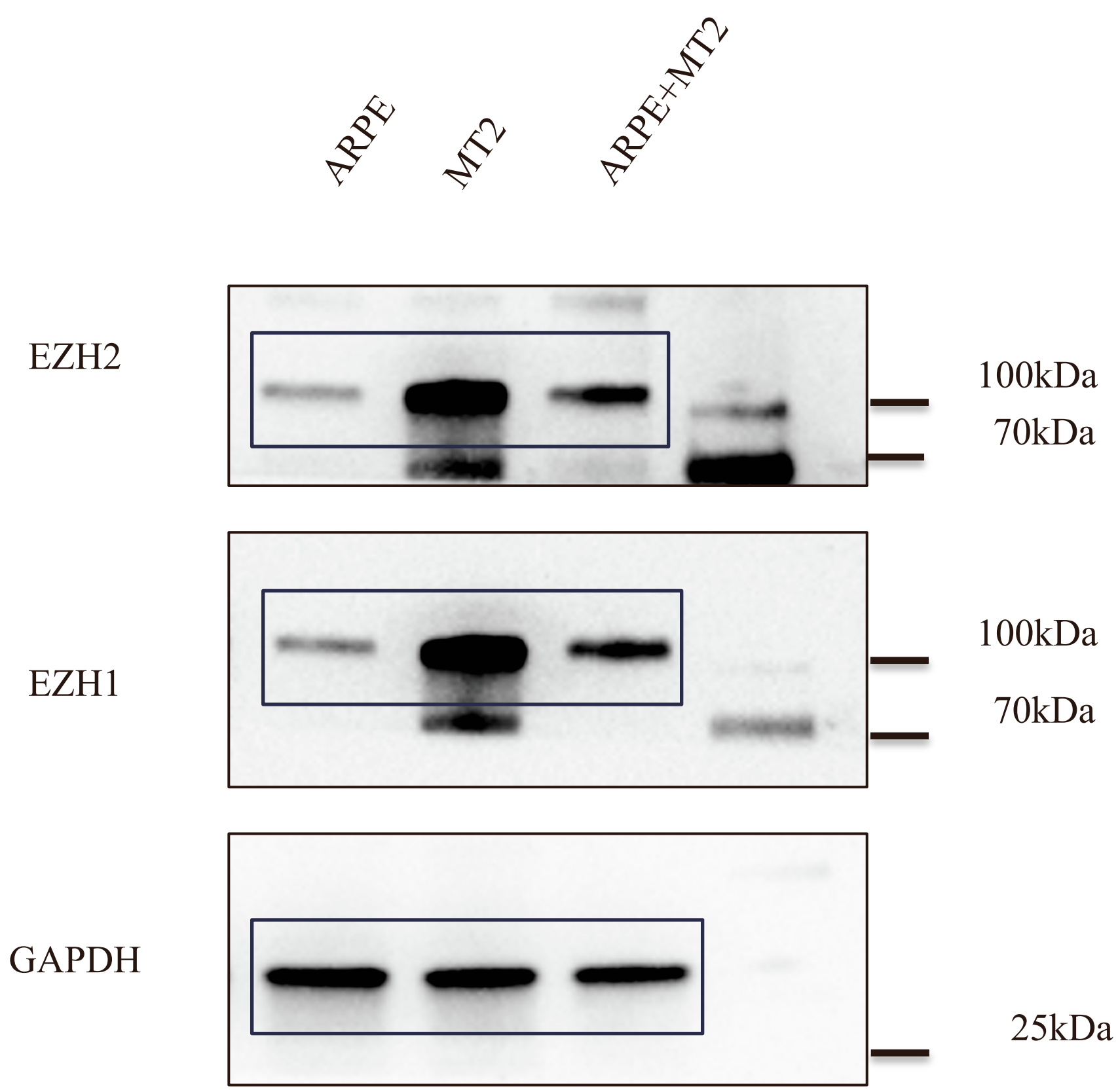

Figure 1D

Whole membrane

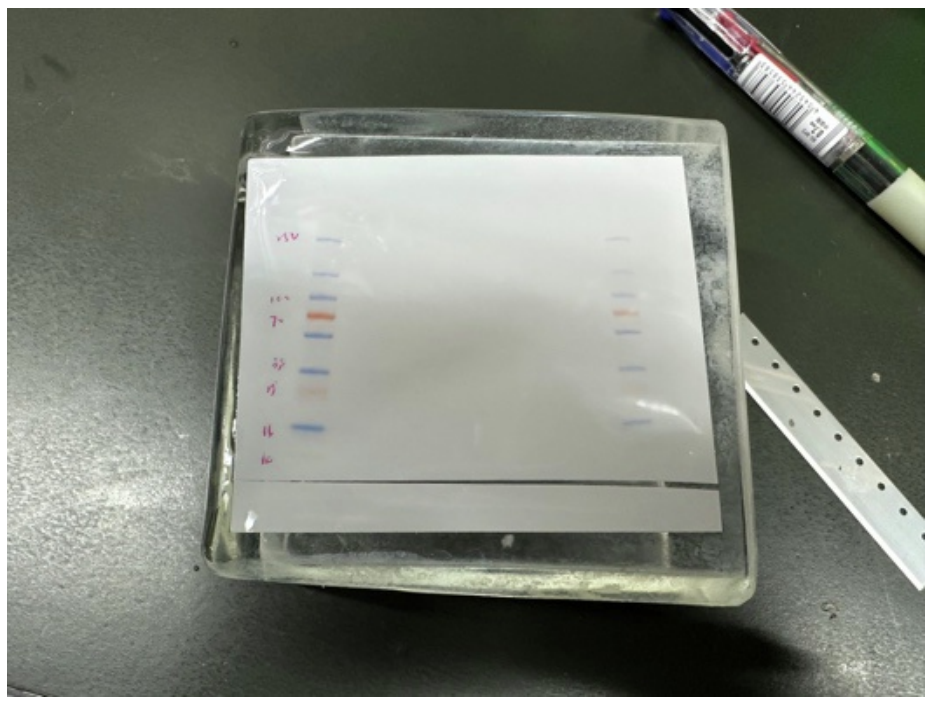

Expose

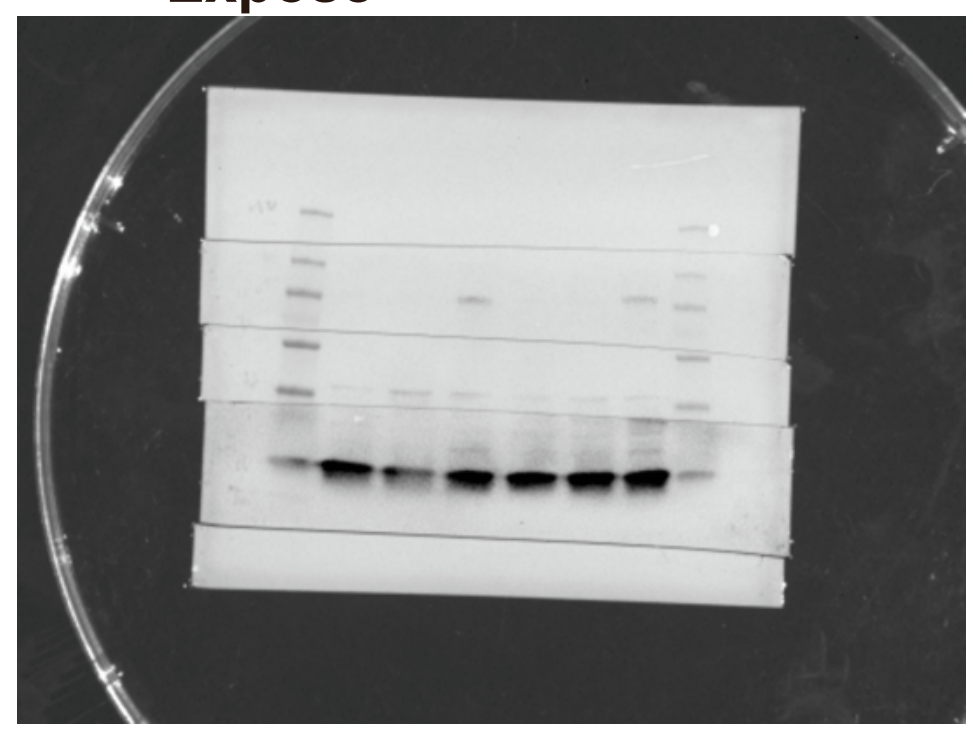

H3K27me3

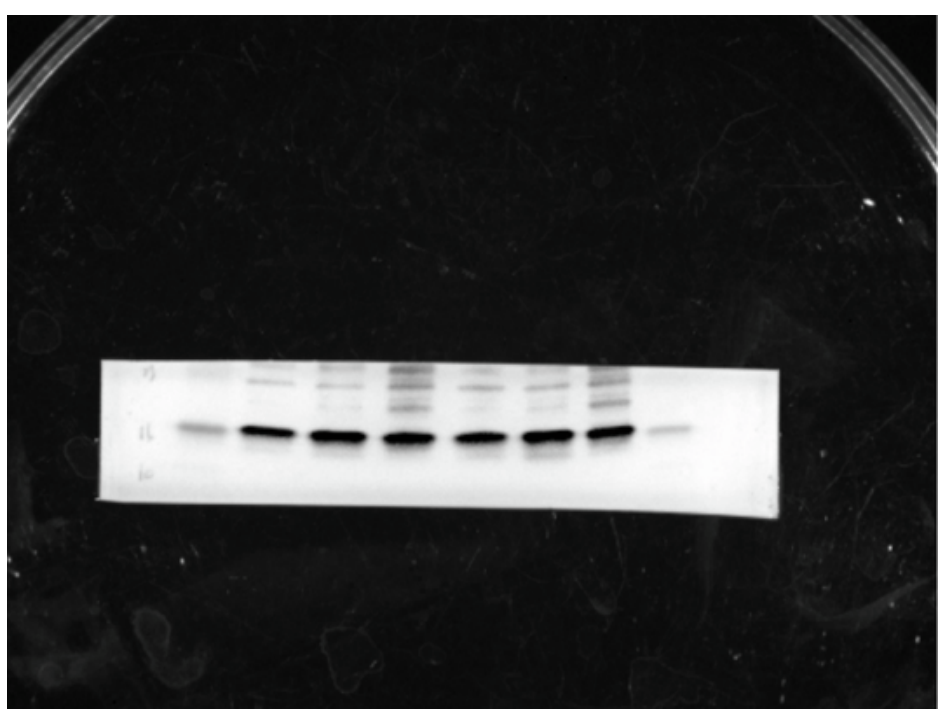

Histone H3

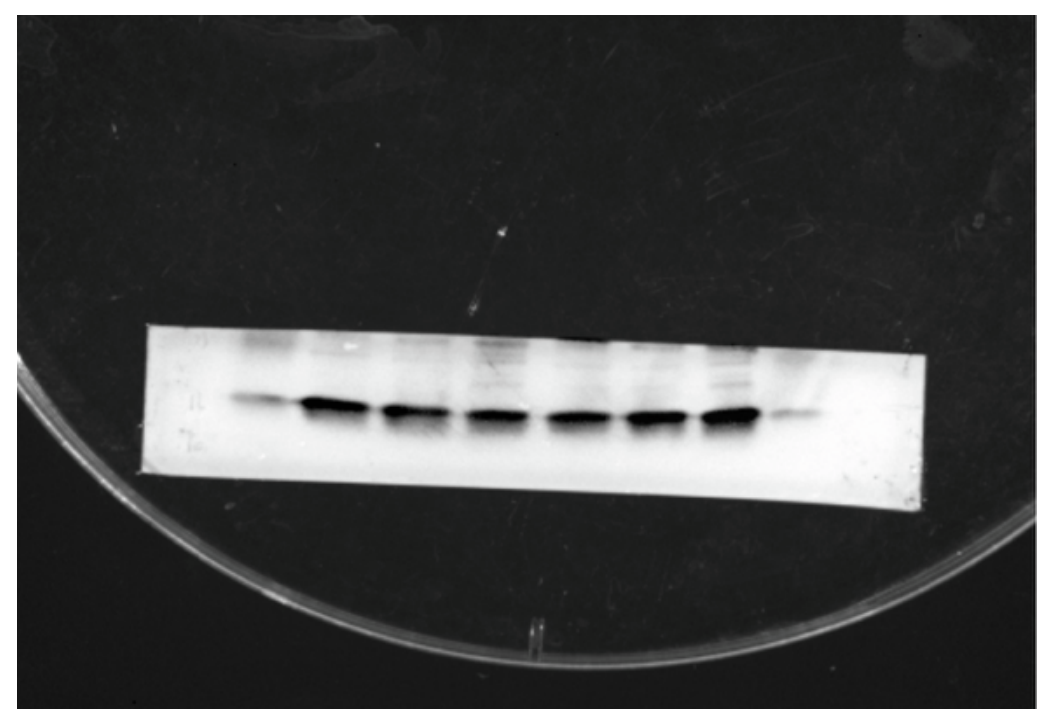

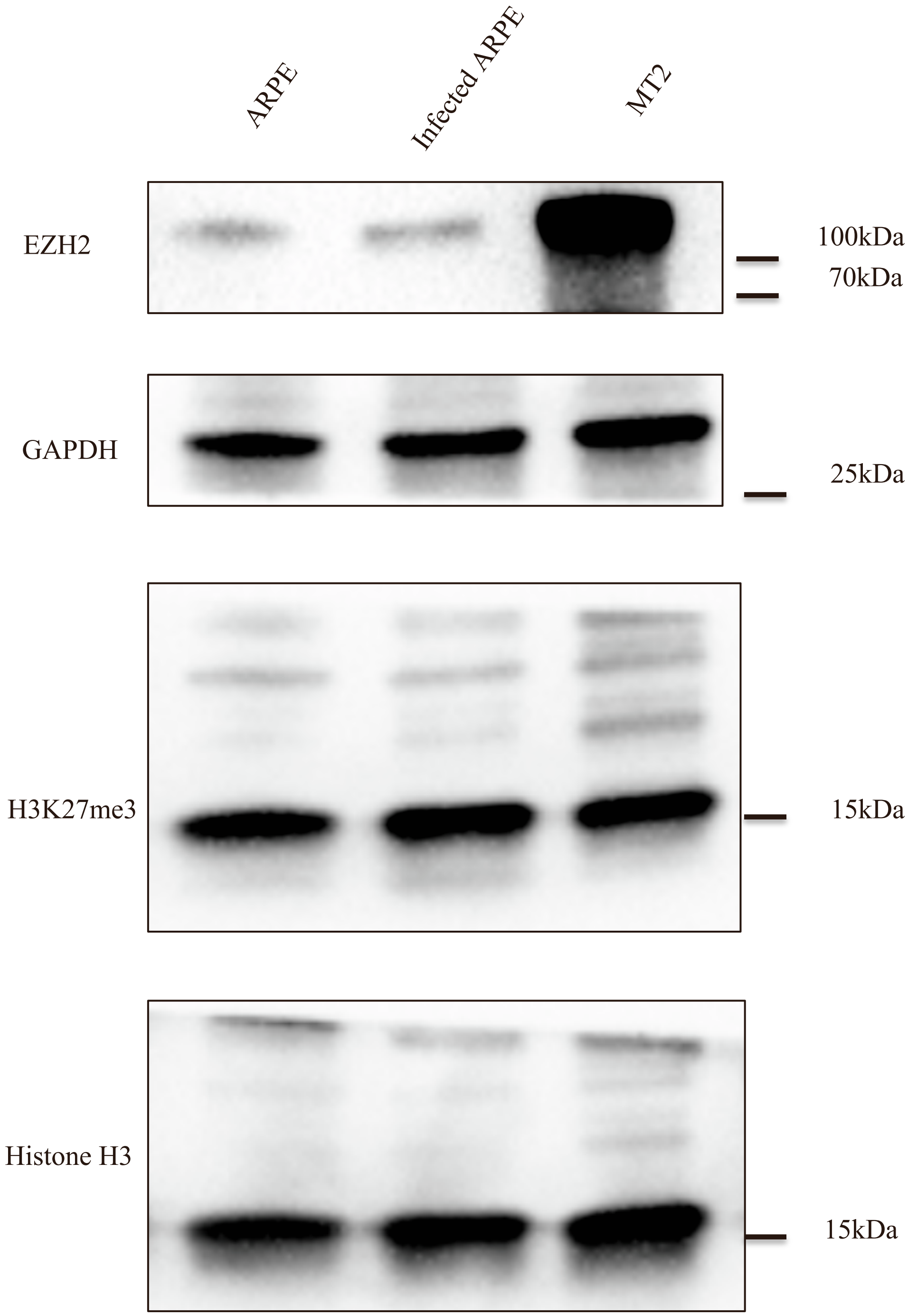
ARPE
Infected ARPE
MT2
EZH2
100kDa
70kDa
GAPDH
25kDa
H3K27me3
15kDa
Histone H3
15kDa
